# Cost of Serious Infections in Chronic Lymphocytic Leukemia

**DOI:** 10.1002/cam4.71397

**Published:** 2026-02-03

**Authors:** Sara Carrillo de Albornoz, Rainier Arnolda, Alisa M. Higgins, Erica M. Wood, Zoe K. McQuilten, Dennis Petrie

**Affiliations:** ^1^ School of Public Health and Preventive Medicine Monash University Melbourne Australia; ^2^ Centre for Health Economics Monash University Melbourne Australia; ^3^ Department of Clinical Haematology Monash Health Melbourne Australia; ^4^ Department of Haematology Alfred Health Melbourne Australia

**Keywords:** chronic lymphocytic leukemia, cost, immunoglobulin, infections

## Abstract

**Background:**

The economic burden of chronic lymphocytic leukemia (CLL) is high, and is projected to increase with the introduction of new targeted treatments and improved survival. These high costs are not only associated with anticancer treatment, but also with the treatment and prevention of CLL symptoms and adverse events. Infections are among the most common adverse events in CLL patients, resulting from immune dysregulation caused by both the underlying disease and treatments. Immunoglobulin replacement therapy (IgRT) is one prophylactic measure used to prevent infections, but its effectiveness in CLL is unclear and costs are substantial. The aim of this paper was to estimate the excess cost associated with serious infections in patients with CLL, and explore other factors that may increase hospitalization costs in Australia.

**Methods:**

We conducted a retrospective longitudinal study of linked hospital data, including 3705 patients with CLL and hospital admissions between July 2016 and June 2022. We estimated the excess cost associated with serious infections, inhospital anticancer treatment and IgRT using generalized linear models with gamma distribution and identity link.

**Results:**

Over the study period, the mean inhospital cost per patient per month was AU$1291 (US$892) and was highest in the month of CLL diagnosis, at AU$4168 (US$2880). The excess cost in the month of a serious infection was AU$22,905 (US$15,829) per patient, and costs remained higher in the subsequent 6 months. The monthly costs associated with IgRT and anticancer treatment were AU$3288 (US$2772) and AU$5223 (US$3609) per patient, respectively.

**Conclusion:**

Our results highlight the high economic burden of serious infections in a large cohort of patients with CLL over a 6‐year period. Further costing studies including costs to the patient and healthcare utilization in the outpatient setting are needed to ascertain the total cost of infections and the overall cost of cancer care in patients with CLL.

## 1 Introduction

Chronic lymphocytic leukemia (CLL) is one of the most common hematological malignancies [1, 2]. The incidence of CLL has been increasing worldwide since the 1990s [3], along with survival due to advances in treatment [4]. The overall economic burden of CLL is high and variable, with annual direct costs ranging from US$4491 in Germany to US$43,913 in the US [5]. These costs are not only related to anticancer treatment, but also to the management of CLL symptoms and treatment‐related side effects.

Immune dysregulation is present from the early stages of CLL and worsens with disease progression and treatment [6, 7, 8]. This makes patients with CLL susceptible to infections, which are a major contributor to morbidity and mortality, and also a key driver for healthcare utilization and costs [9, 10, 11]. The incidence of infections can be affected by the types of anticancer therapies and lines of treatment [12]. The introduction of targeted therapies in CLL has improved survival, but also increased the risk of certain types of infections [7, 8, 12, 13]. Recent US studies have reported healthcare costs per patient per year of US$54,658, which rise with subsequent lines of treatment [14]. Moreover, monthly per‐patient costs also increased with a higher number of adverse events, of which infection is one of the most frequently observed [15, 16].

Despite the high clinical and economic burden of infections in these patients, there are currently no standard guidelines for infection prophylaxis in CLL [12]. Understanding the cost of infections in this population is essential to inform guidelines on the most appropriate interventions to prevent infections in CLL. Immunoglobulin replacement therapy (IgRT) is one of the prophylactic measures used to prevent infections, and some guidelines recommend its use in CLL patients with severe hypogammaglobulinemia (low immunoglobulin G) and recurrent or severe infections [17]. However, the cost of IgRT is also substantial [18] and the impact on infection prevention is uncertain, and may be changing with the advent of a range of new targeted therapies for CLL [19].

As patients with CLL live longer and receive more treatments over extended periods, the clinical and economic burden associated with infections is likely to increase. Nevertheless, the healthcare costs associated with infectious events and the use of IgRT in CLL remain unclear. The aim of this paper is to estimate the cost of serious infections (defined as infection‐related hospitalizations) in patients with CLL, and explore other factors that may increase hospitalization costs. This is critical information to inform guidelines on the most cost‐effective use of resources for infection prophylaxis.

## 2 Materials and Methods

### 2.1 Study Design and Data Sources

**FIGURE 1:**
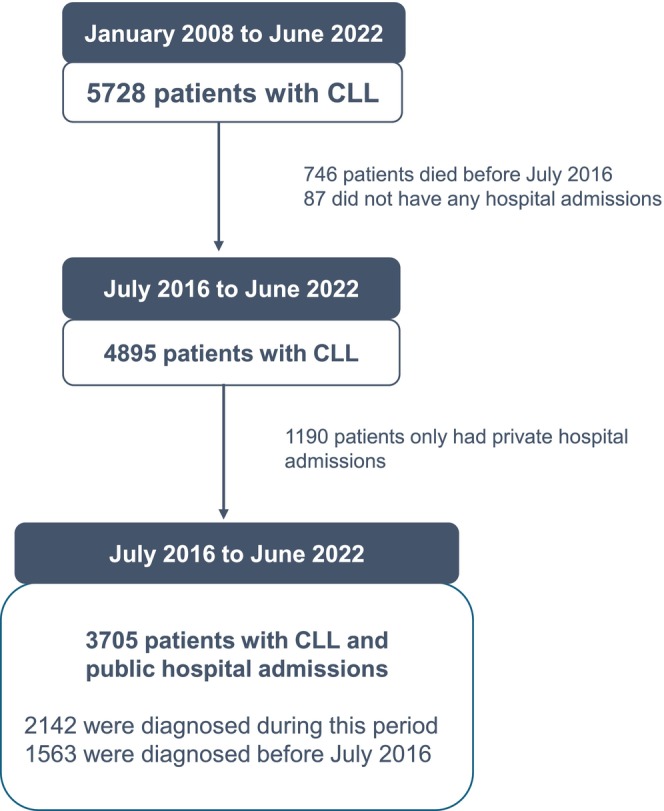
Patient flow. Flow diagram of CLL patients included in this study.

This retrospective cost analysis uses linked longitudinal data from the cancer registry, death registry and administrative hospital datasets in the state of Victoria, Australia. Our cohort included adults (aged 18 years and older) with a new CLL diagnosis in the cancer registry between 1 January 2008 and 31 December 2022, who were alive when costing data became available in the Victorian Cost Data Collection (VCDC), from July 2016 to June 2022 (Figure 1). This analysis included costs and outcomes from the latter period, although patients could have been diagnosed earlier (since January 2008).

CLL patients were identified via the Victorian Cancer Registry using the ICD‐10‐AM (the WHO International Statistical Classification of Diseases and Related Health Problems, Tenth Revision, Australian Modification), which recorded the date of first CLL diagnosis [20]. In Victoria, reporting of all cancer diagnoses to the Victorian Cancer Registry is mandatory for all healthcare services. The date of death was obtained from the Victorian Death Index. Data on all inpatient hospital admissions were obtained from the Victorian Admitted Episodes Dataset (VAED), where diagnoses are classified using the ICD‐10‐AM and procedures according to the Australian Classification of Health Interventions (ACHI 11th edition) [20]. All ICD‐10‐AM and ACHI codes used are provided in the Tables S2–S5. All patient data were linked to cost data from the VCDC. Data linkage was performed by the Centre for Victorian Data Linkage.

The ethical approval (project ID 40164) waived the requirement to get individual consent for this analysis.

### 2.2 Study Population and Outcome Definitions

We included all patients aged 18 years and older at CLL diagnosis. All patients had at least one hospital admission from July 2016 to June 2022. We excluded patients who had undergone stem cell transplantation, as they are uncommon and likely to have a significantly different infection risk profile. Comorbidities were classified according to the Charlson Comorbidity Index (CCI), which was already calculated in the VAED for each hospital admission. We included any CCI data recorded in the hospital admission dataset in the year prior to diagnosis or at diagnosis. In patients with no hospital admissions in the year prior to or at CLL diagnosis, CCI was not available, and thus classified as “Unknown.” We used the highest CCI for a hospitalization in the year prior to CLL diagnosis. A CCI of 0–2 was classified as “Mild,” 3–4 “Moderate,” and 5 or higher “Severe” [21]. Although the cost‐analysis study period was 2016 and 2022, age, sex, and CCI were captured for all of the included patients at the time of diagnosis, between 2008 and 2022. The variable “time from diagnosis” was created to explore the impact of a longer disease duration on hospital costs, thus accounting for any temporal mismatch between patients diagnosed before cost data became available (from January 2008 to July 2016) and those diagnosed during the cost data period.

The ICD‐10‐AM codes were used to identify CLL diagnoses in the cancer registry and infection‐related hospitalizations in the VAED (Tables S5 and S8). ACHI procedure codes were used to identify IgRT, inhospital anticancer treatment and stem cell transplant procedures (Table S6) in the VAED. These procedures were assigned to the patient when the relevant ACHI code appeared in a given admission. Australian refined diagnosis‐related groups (AR‐DRGs) that identified infections were compared to and added to the ICD‐10‐AM infection codes for completeness (Table S7). Serious infections were defined as multi‐day infection‐related hospitalizations with an ICD‐10 and/or AR‐DRG infection code. We used the ICD‐10‐AM infection codes from a published sepsis study in Victoria [22], and these were further adapted to capture serious infections in our target population based on consultation with clinical experts. Only parenteral anticancer treatment administered in hospital was included. We did not include oral anticancer treatments as the majority of these are given in the outpatient setting, which was not available in our dataset. The hospital procedure code for IgRT only included intravenous Ig (IVIg), as administration of subcutaneous Ig (SCIg) was not recorded in the VAED. Prior to 2022, relatively little SCIg was used in Victoria for this indication [23].

### 2.3 Estimating Hospital Care Costs

The cost analysis was undertaken from a public hospital perspective. Only public hospital costs were included in this analysis, as cost data were not supplied by private hospitals. In Australia, the public health system covers the full cost of admissions to public hospitals through Medicare, with no out‐of‐pocket cost to the patient, while private healthcare insurance reimburses private hospital treatments with different levels of coverage and potential out‐of‐pocket costs [24]. Patients with private healthcare insurance in Australia can choose to attend private or public hospitals.

Expenses directly relating to the delivery of patient care were defined as direct costs, while non‐patient related expenses were referred to as overhead or indirect costs [25]. Pharmacy costs were included within the direct costs. Indirect costs to the patient (e.g., out‐of‐pocket costs, productivity loss) were not available. IgRT and inhospital anticancer drugs administered within public hospitals are subsidized by the Australian government, at no direct cost to patients.

All costs were adjusted to 2024 Australian dollars (AU$) using the consumer price index for medical and hospital services published by the Australian Bureau of Statistics (September 2024 quarter) [26]. Costs were converted to 2024 US dollars (US$) using the EPPI‐Centre cost converter and included in the discussion to allow comparability with other studies [27].

### 2.4 Statistical Analysis

Panel data of CLL patients with public hospital admissions were used for this cost analysis. Descriptive statistics are presented for all patients (Table 1). The available follow‐up period for each CLL patient was calculated as the time from July 2016 or CLL diagnosis date (for patients diagnosed after July 2016) until the last available date (June 2022 or death). The annual mean number of any‐cause hospital admissions, serious infections, and admission episodes to receive IgRT or inhospital anticancer treatment was estimated per patient per year of follow‐up.

**TABLE 1:** Key characteristics of CLL patients included in the costing analysis.

Number of patients	3705
Time from diagnosis to first admission during the costing period	
1 year	2511 (67.8%)
1–5 years	809 (21.8%)
5–10 years	385 (10.4%)
Age at CLL diagnosis	
Younger than 60 years	657 (17.7%)
60–69 years	992 (26.8%)
70 years and older	2056 (55.5%)
Sex	
Male	2383 (64.3%)
CCI at diagnosis	
Unknown	1450 (39.1%)
Mild	1910 (51.6%)
Moderate	220 (5.9%)
Severe	125 (3.4%)
Follow‐up (years); mean (SD), median (IQR range)	3.8 (1.9), 4.1 (2.2, 5.9)
Number of admissions per year of follow‐up, mean (SD)	3.7 (8.3)
Serious infections	
Any infections during follow‐up, *n* (%)	1689 (45.6%)
Serious infections per year of follow‐up, mean (SD)	0.5 (1.4)
IgRT	
Any IgRT during follow‐up, *n* (%)	332 (9.0%)
IgRT episodes per year of follow‐up, mean (SD)	0.4 (1.8)
Inhospital Anticancer treatment	
Any anticancer treatment during follow‐up, *n* (%)	972 (26.2%)
Anticancer treatment per year of follow‐up, mean (SD)	0.8 (2.6)
Died during follow‐up, *n* (%)	1056 (28.5%)

*Note:* Serious infections were defined as multi‐day infection‐related hospitalizations with an ICD‐10 and/or AR‐DRG infection code. IgRT or anticancer episodes per year of follow‐up refer to hospital admissions to receive IgRT or anticancer treatment.

Abbreviations: CCI, Charlson Comorbidity Index; CLL, chronic lymphocytic leukemia; IgRT, immunoglobulin replacement therapy; IQR, interquartile range; SD, standard deviation.

**FIGURE 2:**
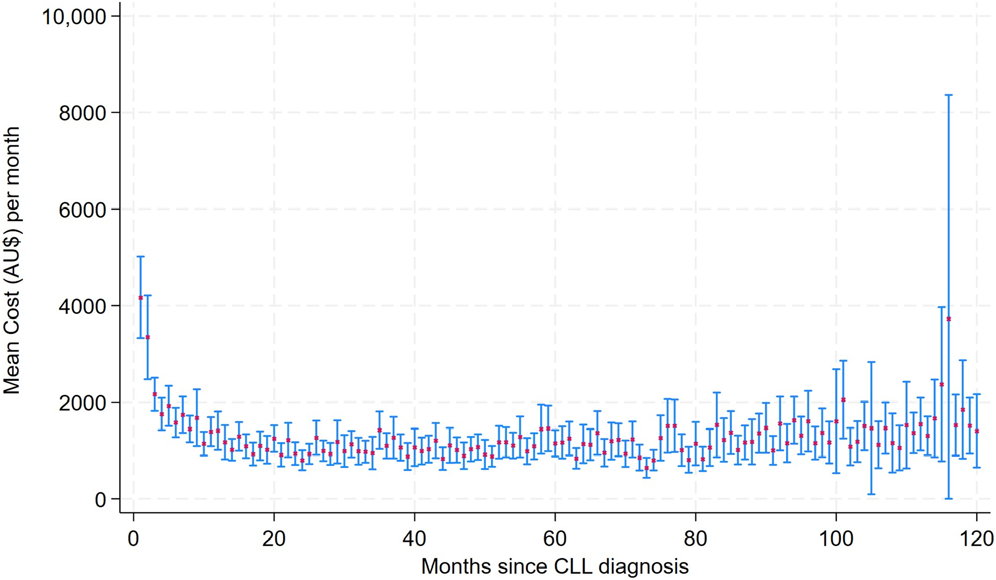
Mean cost (AU$) per patient per month and 95% CI during the follow‐up, according to time since CLL diagnosis. CLL diagnosis dates ranged from January 2008 to June 2022. The follow‐up period with cost data was July 2016 to June 2022. The mean cost per patient per month during the follow‐up was AU$1291.

The costs incurred by each patient during each month were summed to determine their monthly hospital costs. If a patient did not use inhospital services that month, the cost was zero. The mean cost per patient per month was estimated according to time (months) since diagnosis at each episode (Figure 2). Patients were censored after the month they died.

We predicted those factors associated with excess monthly public hospital costs using generalized linear regression models (GLMs) [28]. Excess costs referred to the contribution to mean costs from each of the relevant factors. We considered six different functional forms for the GLMs using three distribution families (Gaussian, Poisson, Gamma) and two link functions (identity and natural log). The model with the best fit was selected using the Modified Park's test and the Pregibon link test (Table S1) [29]. The mean monthly costs associated with serious infections and other events, adjusted for baseline characteristics (e.g., age, sex, CCI), time since diagnosis, inhospital anticancer treatment and IgRT use, were calculated using a GLM model with gamma distribution and identity link. Three separate GLM regressions were conducted to estimate the association with total costs (Table 2), direct costs and indirect costs (Table S2). A categorical variable representing the period impacted due to the COVID pandemic (March 2020 up to June 2022 [30]) was included in the analysis to assess any potential impact on costs during this period. A sensitivity analysis was conducted in a subset of 2051 patients who never had private hospital admissions (Table S4) to examine the potential effect of removing patients with both private and public hospital admissions on costs. Furthermore, descriptive statistics were presented for all CLL patients (Table S3), according to hospital admission type, before the removal of private admissions.

**TABLE 2:** Key factors associated with excess total costs (AU$) per patient per month.

	Monthly hospital cost (95% CI)
Serious infection	22,905 (15,299, 30,512)
1–3 months after index infection	1423 (974, 1871)
4–6 months after index infection	195 (46, 343)
Anticancer treatment	5223 (3751, 6696)
IgRT	3288 (2356, 4219)
CCI (ref. Unknown)	
Mild	88 (58, 118)
Moderate	289 (163, 416)
Severe	515 (224, 807)
Age (ref. < 60 years)	
60–69	−11 (−47, 26)
70 years and older	81 (43, 119)
Sex (ref. Male)	
Female	−71 (−100, −42)
Covid period (March 2020–June 2022)	27 (−3, 56)
Time from diagnosis (months)	−0.3 (−0.7, 0.0)
Death	
Month of death	2275 (544, 4006)
1–3 months before death	1951 (1010, 2891)
4–6 months before death	829 (377, 1282)

*Note:* Serious infections were defined as multi‐day infection‐related hospitalizations with an ICD‐10 and/or AR‐DRG infection code. Covid period: March 2020–June 2022. Multivariable generalized linear model with gamma distribution and identity link function adjusted for age, sex, and CCI. Numbers have been rounded. Monthly hospital costs are the model coefficients; no transformation was needed as the link function (identity) was linear. This table includes all the variables in the GLM; no variables were excluded.

Abbreviations: AU$, Australian dollar 2024; CCI, Charlson Comorbidity Index; CI, confidence interval; IgRT, immunoglobulin replacement therapy.

## 3 Results

Our cohort included 3705 patients with CLL and public hospital admissions between July 2016 and June 2022 (Figure 1), although many were diagnosed earlier (from January 2008). Patients with only private hospital admissions were excluded. Among the included patients, 67.8% of admissions were public and 32.2% were private, and the private hospital admissions were excluded for the cost analysis.

Time from diagnosis to the first public hospital admission during the cost‐analysis period (2016–2022) is presented in Table 1. The majority of patients were male (64.3%), and over half were aged 70 years or older (55.5%) and presented with mild comorbidities (51.6%) in the year prior to their CLL diagnosis. At the first public hospital admission during the cost‐analysis period, 67.8% of patients had been diagnosed with CLL within a year, 21.8% in the previous 1–5 years, and 10.4% had their CLL diagnosis over 5 years before. Over the mean follow‐up of 3.8 years, 45.6% of patients experienced at least one serious infection, 9% received IgRT, 26.2% had inhospital anticancer treatment, and 28.5% died. The mean number of public hospital admissions per patient per year was 3.8, the mean number of serious infections was 0.5, the mean number of IgRT episodes was 0.4, and admissions to receive inhospital anticancer treatment per patient per year was 0.8.

### 3.1 Mean Inhospital Costs

The mean total inhospital costs per patient per month were AU$1291 (US$892). Figure 2 describes the cost per patient per month according to months since diagnosis. The mean cost per patient per month was highest in the month of diagnosis, AU$4168 (US$2880), which decreased over the next 3 months and remained relatively constant in subsequent years. We observed some higher costs emerging around 8 years after diagnosis, but confidence intervals were large, indicating high uncertainty around these costs.

### 3.2 Inhospital Costs Associated With Serious Infections and Other Factors

Table 2 presents the excess cost per patient per month associated with serious infections, adjusted for baseline characteristics, time from diagnosis, the COVID pandemic period, death, IgRT and inhospital anticancer treatment. The monthly per patient cost in the month of the serious infection was AU$22,905 (US$15,829), and monthly costs continued to be higher in the 6 months following the index infection‐related admission. The total cost associated with a serious infection, in the month of the index infection admission and over the following 6 months, amounted to AU$27,759 (US$19,184) per serious infection.

Other factors associated with higher monthly per patient hospital costs were higher comorbidity burden at diagnosis, male gender, age at diagnosis 70 years or older, inhospital anticancer treatment, and IgRT. IgRT and inhospital anticancer treatment were associated with monthly costs of AU$3288 (US$2772) and AU$5223 (US$3609), respectively. Time from diagnosis was not significantly associated with higher excess costs. Direct costs were the main driver of the excess costs associated with serious infections, at AU$18,124 (US$12,525), while indirect costs were estimated at AU$4785 (US$3306) (Table S2).

After controlling for serious infections, inhospital anticancer treatment and IgRT, costs were not significantly higher in the period of the COVID‐19 pandemic (March 2020–June 2022). Similarly, there were no significant cost differences according to time since diagnosis.

Mean costs per patient per month were higher in the 6 months prior to death, and highest in the month of death, at AU$2275 (US$1572). The total excess costs at the end of life, in the month of death plus the previous 6 months, were AU$10,615 (US$7336) per patient.

A sensitivity analysis restricted to 2051 patients with only public hospital admissions did not considerably impact the excess costs (Supporting Information).

## 4 Discussion

Our study quantified the economic impact of serious infections in a large contemporary population of patients with CLL. The monthly cost of a serious infection was considerable and remained high for 6 months after the event. In addition, the cost of IgRT given to prevent infections was associated with higher monthly costs. Other factors associated with excess monthly costs were age older than 70 years and comorbidities at diagnosis, and inhospital anticancer therapy.

CLL is one of the hematological malignancies with the highest use of IgRT [31]. In Australia IgRT use in CLL has been steadily increasing since 2013, with only a slight decline from 2020 to 2022 [32]. Therefore, providing an estimate of the cost of IgRT using real‐world evidence is key to help optimize resource allocation in the health system and improve patient care. In our study, IgRT was associated with an excess cost per patient per month of AU$3288 (US$2272). As patients with CLL are usually treated with IgRT once per month, a full year of IgRT would add up to AU$39,456 (US$27,267). Given our estimated cost of serious infections, the cost of ongoing IgRT would be offset if it prevents 1.4 serious infections per patient per year. Nevertheless, less than half of the patients (45.6%) in our cohort had an infection‐related admission during the follow‐up; the mean number of serious infections per patient per year was 0.5, and only 9% received IgRT. Therefore, careful selection of patients at high risk of repeated serious infections may need to be considered in this population before starting IgRT.

Mean hospital costs (AU$1291 per patient per month, or AU$15,492 per patient per year) were similar to those previously estimated in a small feasibility study of patients with hematological malignancies in Australia (AU$10,000 per patient per year) [18]. Mean costs were highest in the first month of diagnosis and gradually declined over the following 6 months, remaining relatively constant for future years. This indicates a higher healthcare utilization and economic burden at an early stage, and it is consistent with population‐based studies of cancer patients in Canada and Australia, where mean monthly healthcare costs were higher in the month of cancer diagnosis and declined in subsequent months [33, 34]. At the same time, we found excess costs at the end of life of AU$2275 in the last month, which increased over AU$10,000 when considering the previous 6 months before death. U‐shaped excess costs per person per month were described in a New Zealand study of cancer patients, including all cancer types, according to time since diagnosis; highest in the first month post‐diagnosis and rising again in the last month of life [35]. A gradual rise in costs has been reported in cancer patients in the 12 months before death, with a rapid increase in the final 2–3 months [34]. In patients with metastatic cancer, higher costs in the last 6 months of life were found to be driven by inpatient admissions and longer hospital stays [36]. Inaccurate prognostic beliefs and preference for life extension were associated with a higher likelihood of high‐intensity hospital admissions, and integration of palliative and oncology care was suggested as a strategy to reduce hospitalizations at the end of life [36].

It is challenging to compare published healthcare costs between countries due to differences in health systems. Most of the economic literature in CLL has explored the cost‐effectiveness of specific anticancer treatments [5, 37]. The systematic review by Frey et al. [5] included five studies reporting annual direct healthcare costs in CLL, and showed that cost per patient per year varied widely across studies conducted in different countries. In a recent US study, Goyal et al. estimated the mean cost for inpatient admissions as US$2066 per patient per month in CLL patients receiving at least one systemic therapy [15]. This cost is higher than our mean inhospital costs per patient per month of $1291 (US$892), probably explained by the relatively low proportion (26.2%) of patients receiving inhospital anticancer treatment during our study period. Nevertheless, higher healthcare costs in general in the US might also contribute to this difference, as cancer expenditure per capita in the US is almost double that of Australia (cost per capita US$584 vs. US$304, respectively) [38].

In contrast, the cost per patient per month for hospital admissions related to infection reported by Goyal et al. was lower than our estimate. The authors calculated costs per patient per month associated with infections during first‐line anticancer treatment, ranging from US$632 for patients receiving bendamustine plus rituximab to US$1266 for ibrutinib [15]. In our study, the mean cost per patient per month associated with a serious infection was $22,905 (US$15,829). This difference may be due to the methodological approaches used in the cost analysis, as Goyal et al. only measured costs associated with the first infection experienced during their first line of active treatment, while we included repeated infections during the whole follow‐up period. In their study [15], fewer than 5% of patients had infections during active treatment, whereas more than 50% had infections before anticancer treatment was initiated. When the authors explored the costs associated with multiple adverse events (of which infections were the most common non‐hematologic adverse events after hypertension) during the full follow‐up period, mean costs per patient per month increased with the number of adverse events and lines of treatment [15].

The monthly cost associated with inhospital anticancer treatment in our study was AU$5223 (US$3609). Most patients in our cohort (73.8%) did not receive any anticancer therapy in hospital, although they could have been treated with oral targeted therapies (e.g., ibrutinib or venetoclax) in the outpatient setting, which may have changed their infection risk profile and increased the overall cost of treatment. However, the number of patients receiving oral targeted therapies in the state of Victoria during the study period was likely small, given that ibrutinib was only listed in Australia for the treatment of CLL in December 2017, and the average number of patients receiving it per year after its introduction was 1500 across the whole of Australia. This number then plateaued to 1000 per year after the listing of venetoclax in March 2019, which acquired part of the market share [39].

A greater comorbidity burden at CLL diagnosis was also associated with higher monthly costs, with patients with a severe CCI score (5 or higher) having 6 times higher monthly costs than those with mild CCI scores (0–2) when compared to those that had not been to hospital in the 12 months prior to their diagnosis. This is consistent with previous costing studies in CLL patients [15]. Healthcare resource utilization has been reported to significantly increase with the number of comorbidities in CLL, suggesting multidisciplinary care plans may be needed for patients with CLL and concomitant conditions [40].

Our study has several limitations. Firstly, it is a retrospective study that used administrative data. The lack of some key clinical prognostic factors, disease severity and out‐of‐hospital anticancer treatments, as well as the presence of other unknown confounding factors could have had an impact on the estimated costs. It is important to note that these results do not imply causal relationships; these analyses explored the association and contribution of the presented factors to the monthly hospital costs, but there remains a possibility that unobserved confounding factors may also contribute to these cost estimates.

The use of ICD‐10 codes and AR‐DRGs to identify serious infections also raises the possibility of overestimating inhospital infection events, if the codes relate to infections in previous admissions rather than the current admission. We limited the possibility of coding errors by restricting the definition of serious infections to multi‐day admissions, and removing infection‐related admissions that only lasted 1 day. Furthermore, high quality of data coding has been previously demonstrated in administrative hospital data in the state of Victoria [41].

Our analysis only included inhospital admissions, and outpatient anticancer treatment, infections outside of hospital and oral antibiotic treatments outside of hospital were missing. However, inpatient costs have been shown to be the main cost drivers in CLL. The low utilization of oral targeted therapies in Australia during the study period [39] suggests the absence of oral anticancer treatments in our analysis was unlikely to substantially affect our results; however, as the uptake of oral treatments increases, the impact on healthcare costs may also rise. Further research including outpatient costs is needed to estimate the full cost to the healthcare system, beyond the inpatient hospital setting, along with the cost of drug treatments.

Only cost data from public hospitals were available, which potentially underestimates the true cost as some patients could have attended both public and private hospitals during their disease course. In Australia, access to private hospitals is influenced by socioeconomic status and health insurance coverage [42], and patients with private health insurance often go to public hospitals when they need acute care, mainly due to out‐of‐pocket costs for private hospital admissions [43]. An exploratory analysis considering private hospital admissions (Table S3) indicated that patients with only private admissions had a lower comorbidity burden, fewer patients received IgRT or had infection‐related admissions, and mortality during follow‐up was lower than that of patients with only public admissions. Moreover, a sensitivity analysis in patients with only public hospital admissions (Table S4) showed higher costs associated with moderate and severe comorbidities, and in the 6 months preceding death, compared with the full cohort; while the excess costs of serious infections, anticancer treatment, and IgRT were similar to those in the main cost analysis. These results suggest that, despite the potential for underestimating costs associated with elective procedures (e.g., IgRT or anticancer treatment) in private hospitals, patients admitted to public hospitals had a higher level of morbidity and mortality, which were associated with increased monthly costs. Overall, the low proportion of private hospital admissions and the full funding of both Ig product and administration in the public health system suggest that the inclusion of private hospital costs may not considerably alter these cost estimates.

In addition, we could not explore indirect costs to the patients and productivity losses. Patients diagnosed with cancer in the past 2 years are more likely to incur significantly higher (> AU$10,000) out‐of‐pocket healthcare expenses than patients without cancer [44]. A key strength of our study is the linked dataset that captures all patients with CLL and their public hospital costs in the state of Victoria, providing contemporary real‐world evidence of the economic burden of CLL to the public health system. The large cohort of patients with CLL and the measurement of repeated serious infections during a long follow‐up provides important evidence that highlights the substantial economic impact that infections have on the hospital system. A recently published study using the same dataset explored the association between IgRT and serious infections, indicating that among CLL patients who received regular IgRT, periods of IgRT treatment were not significantly associated with a reduction in serious infections [45]. The high cost of both IgRT and serious infections underscores the need for clinical guidelines in CLL infection prophylaxis, as well as developing strategies to identify patients at highest risk of infections who might benefit the most from IgRT.

In addition, these cost estimates can inform economic models of prophylactic strategies to prevent serious infections and associated costs in this population, and help prioritize the allocation of healthcare resources. Further studies are needed to assess the causal relationship between IgRT, infections, and associated costs in order to estimate the full economic burden of CLL to the patient and the wider society.

## 5 Conclusion

This real‐world study demonstrates the high economic burden of serious infections in a large cohort of patients with CLL over a 6‐year period. Given the increased risk of infection in this population and the high cost of some prophylactic interventions, such as IgRT, these cost estimates highlight the need to identify patients at highest risk and adopt cost‐effective prophylactic strategies. The highest costs were incurred at initial diagnosis and later in the months close to death, which underscores these as key periods in the care pathway where targeted approaches could be established to reduce the economic burden. Further studies including costs to the patient and healthcare utilization in the outpatient setting are needed to fully ascertain the cost of infections and the overall cost of cancer care in patients with CLL.

## Author Contributions

**Sara Carrillo de Albornoz:** conceptualization, methodology, writing – original draft, formal analysis, investigation. **Rainier Arnolda:** writing – review and editing, formal analysis. **Alisa M. Higgins:** methodology, writing – review and editing, supervision. **Erica M. Wood:** writing – review and editing, supervision. **Zoe K. McQuilten:** conceptualization, writing – review and editing, supervision. **Dennis Petrie:** conceptualization, methodology, writing – review and editing, supervision, funding acquisition.

## Ethics Statement

Ethical approval was granted by the Monash University Human Research Ethics Committee (MUHREC), approval number—project ID 40164, and the requirement to get individual consent was waived as these data were obtained from an administrative dataset.

## Conflicts of Interest

Erica Wood and Zoe McQuilten received grant funding from CSL Behring not related to this study and research funding to the institution from Abbvie, Amgen, AstraZeneca, Beigene, Celgene, Janssen, New Zealand Blood Service, Novartis, Sanofi and Takeda. The remaining authors declare no conflicts of interest.

## Supporting information


**Table S1:** Pregibon link test for each GLM.
**Table S2:** Key factors associated with excess direct and indirect costs (AU$) per patient per month.
**Table S3:** Key characteristics of all patients with CLL, according to hospital admission type.
**Table S4:** Key factors associated with excess total costs (AU$) per patient per month (patients with public hospital admissions only).
**Table S5:** ICD‐10‐AM (11th edition, 2019) codes used to define CLL diagnosis.
**Table S6:** ACHI (11th edition, 2019) codes used to define IgRT, stem cell transplant and anticancer treatment.
**Table S7:** AR‐DRGs used to identify chemotherapy and infectious episodes.
**Table S8:** Infectious disease episodes defined using the ICD‐10‐AM (11th edition, 2019).

## Data Availability

The data included in this article were provided via an application process by the Centre for Victorian Data Linkage (CVDL) and are not publicly available. An online application can be made to CVDL via: https://vahi.vic.gov.au/ourwork/data‐linkage/apply.
