# Supplementary material for: Cost of Serious Infections in Chronic Lymphocytic Leukemia
Source: Cancer Med. 2026 Feb 3;15(2):e71397. doi: 10.1002/cam4.71397 (PMC12868929; doi:10.1002/cam4.71397)
Supplement: Supplementary file 1 — Table S1: Pregibon link test for each GLM. Table S2: Key factors associated with excess direct and indirect costs (AU$) per patient per month. Table S3: Key characteristics of all patients with CLL, according to hospital admission type. Table S4: Key factors associated with excess total costs (AU$) per patient per month (patients with public hospital admissions only). Table S5: ICD‐10‐AM (11th edition, 2019) codes used to define CLL diagnosis. Table S6: ACHI (11th edition, 2019) codes used to define IgRT, stem cell transplant and anticancer treatment. Table S7: AR‐DRGs used to identify chemotherapy and infectious episodes. Table S8: Infectious disease episodes defined using the ICD‐10‐AM (11th edition, 2019). [file CAM4-15-e71397-s001.docx]

# **Supplementary materials**

**Title: Cost of serious infections in Chronic Lymphocytic Leukaemia**

Authors: Sara Carrillo de Albornoz, Rainier Arnolda, Alisa M Higgins, Erica M Wood, Zoe K McQuilten, Dennis Petrie

## GLM model selection

Baseline characteristics (age, CCI, sex) and serious infections were included in the regressions to select the most appropriate functional form for the GLM.

The Modified Park’s test was used to identify the family of distribution to use in the GLM: a coefficient close to 0=gaussian, 1=poisson, 2=gamma. For our regression, the coefficient on the Modified Park’s test was 1.6, which is closest to 2, indicating a gamma distribution is likely to be best suited.

Based on Table S2, Identity link with Gamma distribution, was the GLM with the best fit. A significant coefficient in the Pregibon link test indicates an inappropriate link function.

**Table S1. Pregibon link test for each GLM**

| GLM | p-value |
| --- | --- |
| Gamma, Log | NC |
| Gamma, Identity | 0.905 |
| Poisson, Log | NC |
| Poisson, Identity | NC |
| Gaussian, Log | 0.000 |
| Gaussian, Identity | 0.000 |

NC=non-convergent

## Direct and indirect excess costs

The excess direct and indirect costs associated with a serious infection, in the month of the index infection admission and over the following 6 months, amounted to AU$21,973 and $AU5787 per patient, respectively. The excess direct and indirect costs at the end of life, in the month of death and the previous 6 months, were AU$8338 and AU$2299 per patient, respectively.

**Table S2. Key factors associated with excess direct and indirect costs (AU$) per patient per month**

|  | Direct monthly hospital cost (95% CI) | Indirect monthly hospital cost (95% CI) |
| --- | --- | --- |
| Serious infections | 18,123 (12,015, 24,232) | 4785 (3136, 6434) |
| 1-3 months after index infection | 1129 (769, 1490) | 293 (196, 389) |
| 4-6 months after index infection | 154 (34, 273) | 42 (10, 73) |
| Anticancer treatment | 4597 (3297, 5897) | 626 (431, 822) |
| IgRT | 3029 (2174, 3884) | 261 (171, 351) |
| CCI (ref. unknown) |  |  |
| Mild | 72 (47, 96) | 16 (10, 23) |
| Moderate | 232 (130, 335) | 57 (31, 84) |
| Severe | 409 (175, 643) | 105 (42, 168) |
| Age (ref. <60 years) |  |  |
| 60 to 69 | -12 (-42, 18) | 2 (-6, 9) |
| 70 years and older | 63 (32, 95) | 18 (10, 26) |
| Sex (ref. male) |  |  |
| Female | -58 (-81, -35) | -12 (-18, -6) |
| Covid period | 15 (-9, 38) | 12 (6, 18) |
| Time from diagnosis (months) | -0.3 (-0.6, 0.0) | -0.1 (-0.1, 0.0) |
| Death |  |  |
| Month of death | 1787 (410, 3163) | 491 (98, 883) |
| 1-3 months before death | 1519 (778, 2260) | 438 (217, 659) |
| 4-6 months before death | 665 (299, 1031) | 164 (69, 259) |

Multivariable generalized linear model with gamma distribution and identity link function, adjusted for age, sex, and CCI. Numbers have been rounded. Monthly hospital costs are the model coefficients, no transformation was needed as the link function (identity) was linear. This table includes all the variables in the GLM, no variables were excluded.

Serious infections were defined as multi-day infection-related hospitalizations with an ICD-10 and/or AR-DRG infection code.

Covid period: March 2020- June 2022

Abbreviations: AU$: Australian dollar (2024); CCI: charlson comorbidity index; CI: confidence interval; IgRT: immunoglobulin replacement therapy.

## Characteristics of all 4895 CLL patients prior to removal of private hospital admissions

Key patient characteristics in the full cohort of CLL patients before removing private hospital admissions. These data were not included in the cost analysis, but presented to compare the characteristics of CLL patients according to hospital admission type.

**Table S3. Key characteristics of all patients with CLL, according to hospital admission type**

|  | | Private admissions only | Public admissions only | Both public and private admissions |
| --- | --- | --- | --- | --- |
| Number of patients | | 1190 | 2051 | 1654 |
| Time from diagnosis to first admission during the costing period | 1 year | 871 (73.2%) | 1486 (72.5%) | 1025 (62.0%) |
|  | 1-5 years | 215 (18.1%) | 381 (18.6%) | 428 (25.9%) |
|  | 5-10 years | 104 (8.7%) | 184 (9.0%) | 201 (12.2%) |
| Age at CLL diagnosis | Younger than 60 years | 223 (18.7%) | 380 (18.5%) | 276 (16.7%) |
|  | 60 to 69 years | 354 (29.7%) | 513 (25.0%) | 479 (29.0%) |
|  | 70 years and older | 613 (51.5%) | 1158 (56.5%) | 899 (54.4%) |
| Sex | Male | 728 (61.2%) | 1302 (63.5%) | 1073 (64.9%) |
| CCI at diagnosis | Unknown | 485 (40.8%) | 886 (43.2%) | 564 (34.1%) |
|  | Mild | 639 (53.7%) | 951 (46.4%) | 959 (58.0%) |
|  | Moderate | 38 (3.2%) | 132 (6.4%) | 88 (5.3%) |
|  | Severe | 28 (2.4%) | 82 (4.0%) | 43 (2.6%) |
| Follow-up (years); mean (SD) | | 3.7 (2.0) | 3.4 (2.0) | 4.3 (1.7) |
| Number of admissions per year of follow-up, mean (SD) | | 3.9 (8.2) | 4.6 (9.9) | 5.4 (9.9) |
| Serious infections | |  |  |  |
| Any serious infections during follow-up, n (%) | | 305 (25.6%) | 976 (47.6%) | 962 (58.2%) |
| Serious infections per year of follow-up, mean (SD) | | 0.3 (1.0) | 0.6 (1.7) | 0.5 (1.0) |
| IgRT | |  |  |  |
| Any IgRT during follow-up, n (%) | | 90 (7.6%) | 166 (8.1%) | 281 (17.0%) |
| IgRT episodes per year of follow-up, mean (SD) | | 0.4 (2.1) | 0.4 (1.7) | 0.9 (2.7) |
| In-hospital Anticancer treatment | |  |  |  |
| Any anticancer treatment during follow-up, n (%) | | 329 (27.6%) | 585 (28.5%) | 699 (42.3%) |
| Anticancer treatment per year of follow-up, mean (SD) | | 1.4 (5.0) | 1.1 (3.1) | 1.3 (2.6) |
| Died during follow-up, n (%) | | 147 (12.4%) | 643 (31.4%) | 413 (25.0%) |

Serious infections were defined as multi-day infection-related hospitalizations with an ICD-10 and/or AR-DRG infection code.

IgRT or anticancer episodes per year of follow-up refer to hospital admissions to receive IgRT or anticancer treatment.

Abbreviations: CCI: Charlson comorbidity index; CLL: chronic lymphocytic leukemia; IgRT: immunoglobulin replacement therapy; SD: standard deviation

## Total costs in the subgroup of patients (n=2051) with only public hospital admissions

A sensitivity analysis was conducted in 2051 CLL patients who only had public hospital admissions to explore any differences in total costs compared to the full cohort of 3705 CLL patients included in the main cost analysis (Table 2 in the main text).

**Table S4. Key factors associated with excess total costs (AU$) per patient per month (patients with public hospital admissions only) .**

|  | Monthly hospital cost (95% CI) |
| --- | --- |
| Serious infections | 23,972 (14,438, 33,505) |
| 1-3 months after index infection | 1518 (922, 2113) |
| 4-6 months after index infection | 110 (-76, 297) |
| Anticancer treatment | 5124 (3397, 6850) |
| IgRT | 3188 (1970, 4406) |
| CCI (ref. unknown) |  |
| Mild | 63 (12, 114) |
| Moderate | 411 (185, 638) |
| Severe | 947 (324, 1570) |
| Age (ref. <60 years) |  |
| 60 to 69 | 36 (-21, 93) |
| 70 years and older | 158 (100, 217) |
| Sex (ref. male) |  |
| Female | -125 (-173, -78) |
| Covid period | 17 (-29, 63) |
| Time from diagnosis (months) | -0.4 (-1.1, 0.2) |
| Death |  |
| Month of death | 2703 (233, 5174) |
| 1-3 months before death | 2325 (959, 3691) |
| 4-6 months before death | 1013 (336, 1689) |

Multivariable generalized linear model with gamma distribution and identity link function, adjusted for age, sex, and CCI. Numbers have been rounded. Monthly hospital costs are the model coefficients, no transformation was needed as the link function (identity) was linear. This table includes all the variables in the GLM, no variables were excluded.

Serious infections were defined as multi-day infection-related hospitalizations with an ICD-10 and/or AR-DRG infection code.

Covid period: March 2020- June 2022

Abbreviations: AU$: Australian dollar (2024); CCI: charlson comorbidity index; CI: confidence interval; IgRT: immunoglobulin replacement therapy.

## Data sources

**Table S5. ICD-10-AM (11th edition, 2019) codes used to define CLL diagnosis**

| ICD-10 | Description |
| --- | --- |
| CLL diagnosis | |
| C91.1 | Chronic lymphocytic leukaemia B-cell type |

Source: ICD-10-AM Chronicle – First Edition to Eleventh Edition

**Table S6. ACHI (11^th^ edition, 2019) codes used to define IgRT, stem cell transplant and anticancer treatment**

| ICD-10 | Description |
| --- | --- |
| Immunoglobulin | |
| 13706-05 | Administration of gamma globulin |
| Stem cell transplantation | |
| 13706-00 | Allogeneic bone marrow or stem cell transplantation, matched related donor, without in vitro processing |
| 13706-06 | Allogeneic bone marrow or stem cell transplantation, matched related donor, with in vitro processing |
| 13706-07 | Autologous bone marrow or stem cell transplantation, without in vitro processing |
| 13706-08 | Autologous bone marrow or stem cell transplantation, with in vitro processing |
| 13706-09 | Allogeneic bone marrow or stem cell transplantation, other donor, without in vitro processing |
| 13706-10 | Allogeneic bone marrow or stem cell transplantation, other donor, with in vitro processing |
| Anticancer treatment | |
| 9619600 | Intra-arterial administration of pharmacological agent, antineoplastic agent |
| 9619700 | Intramuscular administration of pharmacological agent, antineoplastic agent |
| 9619800 | Intrathecal administration of pharmacological agent, antineoplastic agent |
| 9619900 | Intravenous administration of pharmacological agent, antineoplastic agent |
| 9620100 | Intracavitary administration of pharmacological agent, antineoplastic agent |
| 9620200 | Enteral administration of pharmacological agent, antineoplastic agent |
| 9620500 | Other administration of pharmacological agent, antineoplastic agent |
| 9620600 | Unspecified administration of pharmacological agent, antineoplastic agent |
| 9620700 | Loading of implantable infusion device or pump, antineoplastic agent |
| 9620800 | Loading of ambulatory drug delivery device, antineoplastic agent |
| 9620900 | Loading of drug delivery device, antineoplastic agent |

Source: Australian Classification of Health Interventions (ACHI) Chronicle – First Edition to Eleventh Edition

**Table S7. AR-DRGs used to identify chemotherapy and infectious episodes**

| AR-DRG | Description |
| --- | --- |
| R63Z | Chemotherapy |
| T01A | Infectious and Parasitic Diseases W GIs, Major Complexity |
| T01B | Infectious and Parasitic Diseases W GIs, Intermediate Complexity |
| T01C | Infectious and Parasitic Diseases W GIs, Minor Complexity |
| T40Z | Infectious and Parasitic Diseases W Ventilator Support |
| T60A | Septicaemia, Major Complexity |
| T60B | Septicaemia, Intermediate Complexity |
| T60C | Septicaemia, Minor Complexity |
| T61A | Postoperative Infections, Major Complexity |
| T61B | Postoperative Infections, Minor Complexity |
| T62A | Fever of Unknown Origin, Major Complexity |
| T62B | Fever of Unknown Origin, Minor Complexity |
| T63A | Viral Illnesses, Major Complexity |
| T63B | Viral Illnesses, Minor Complexity |
| T64B | Other Infectious and Parasitic Diseases, Intermediate Complexity |
| T64C | Other Infectious and Parasitic Diseases, Minor Complexity |

Source: National Hospital Cost Data Collection, AR-DRG version 10.0, Round 24 (2019-20)

**Table S8. Infectious disease episodes defined using the ICD-10-AM (11th edition, 2019)**

| ICD-10 | Description |
| --- | --- |
| CERTAIN INFECTIOUS AND PARASITIC DISEASES | |
| A00.0 | Cholera dt 01 biovar cholerae |
| A00.1 | Cholera dt biovar eltor |
| A00.9 | Cholera unspecified |
| A01.0 | Typhoid fever |
| A01.1 | Paratyphoid fever A |
| A01.2 | Paratyphoid fever B |
| A01.3 | Paratyphoid fever C |
| A01.4 | Paratyphoid fever unspecified |
| A02.0 | Salmonella enteritis |
| A02.1 | Sepsis due to Salmonella |
| A02.2 | Localised Salmonella infections |
| A02.8 | Other specified Salmonella infections |
| A02.9 | Salmonella infection unspecified |
| A03.0 | Shigellosis due to Shigella dysenteriae |
| A03.1 | Shigellosis due to Shigella flexneri |
| A03.2 | Shigellosis due to Shigella boydii |
| A03.3 | Shigellosis due to Shigella sonnei |
| A03.7 | Sepsis due to Shigella |
| A03.8 | Other shigellosis |
| A03.9 | Shigellosis unspecified |
| A04.0 | Enteropathogenic E coli infection |
| A04.1 | Enterotoxigenic E coli infection |
| A04.2 | Enteroinvasive E coli infection |
| A04.3 | Enterohaemorrhagic E coli infection |
| A04.4 | Other E coli infection |
| A04.5 | Campylobacter enteritis |
| A04.6 | Enteritis due to Yersinia enterocolitica |
| A04.7 | Enterocolitis due to C.difficile |
| A04.8 | Other spec bacterial intestinal infectn |
| A04.9 | Bacterial intestinal infection unsp |
| A05.0 | Food-borne staphylococcal intoxication |
| A05.1 | Botulism |
| A05.2 | Food-borne intox dt C. perfringens |
| A05.3 | Food-borne intox dt Vib parahaemolyticus |
| A05.4 | Food-borne Bacillus cereus intoxication |
| A05.8 | Other spec bacterial food-borne intox |
| A05.9 | Bacterial food-borne intoxication unsp |
| A06.0 | Acute amoebic dysentery |
| A06.1 | Chronic intestinal amoebiasis |
| A06.2 | Amoebic nondysenteric colitis |
| A06.3 | Amoeboma of intestine |
| A06.4 | Amoebic liver abscess |
| A06.5 | Amoebic lung abscess |
| A06.6 | Amoebic brain abscess |
| A06.7 | Cutaneous amoebiasis |
| A06.8 | Amoebic infection of other sites |
| A06.9 | Amoebiasis unspecified |
| A07.0 | Balantidiasis |
| A07.1 | Giardiasis [lambliasis] |
| A07.2 | Cryptosporidiosis |
| A07.3 | Isosporiasis |
| A07.8 | Other spec protozoal intestinal diseases |
| A07.9 | Protozoal intestinal disease unsp |
| A08.0 | Rotaviral enteritis |
| A08.1 | Acute gastroenteropathy dt Norovirus |
| A08.2 | Adenoviral enteritis |
| A08.3 | Other viral enteritis |
| A08.4 | Viral intestinal infection unspecified |
| A08.5 | Other specified intestinal infections |
| A09.0 | Oth gastroenteritis & colitis infect |
| A09.9 | Gastroenteritis & colitis unsp origin |
| A15.0 | TB lung conf sputm mico w or wo cult |
| A15.1 | TB lung confirmed by culture only |
| A15.2 | TB lung confirmed histologically |
| A15.3 | TB lung confirmed by unspecified means |
| A15.4 | TB intrathoracic nodes conf bact & histo |
| A15.5 | TB larynx trach bronc conf bact & histo |
| A15.6 | Tuberculous pleurisy conf bact & histo |
| A15.7 | Primary respiratory TB conf bact & histo |
| A15.8 | Other respiratory TB conf bact & histo |
| A15.9 | Respiratory TB unsp conf bact & histo |
| A16.0 | TB lung negative bact & histo |
| A16.1 | TB lung no bact & histo exam done |
| A16.2 | TB lung wo bact & histo confirmation |
| A16.3 | TB intrathor nodes wo bact & histo conf |
| A16.4 | TB lrnx trach bronc wo bact & histo conf |
| A16.5 | Tubrcul pleurisy wo bact & histo conf |
| A16.7 | Primary resp TB wo bact & histo conf |
| A16.8 | Oth respiratory TB wo bact & histo conf |
| A16.9 | Respiratory TB unsp wo bact & histo conf |
| A17.0 | Tuberculous meningitis |
| A17.1 | Meningeal tuberculoma |
| A17.8 | Other tuberculosis of nervous system |
| A17.9 | TB of nervous system unsp |
| A18.0 | Tuberculosis of bones and joints |
| A18.1 | Tuberculosis of genitourinary system |
| A18.2 | Tuberculous peripheral lymphadenopathy |
| A18.3 | TB intestine peritoneum mesenteric gland |
| A18.4 | TB skin and subcutaneous tissue |
| A18.5 | Tuberculosis of eye |
| A18.6 | Tuberculosis of ear |
| A18.7 | Tuberculosis of adrenal glands |
| A18.8 | Tuberculosis of other specified organs |
| A19.0 | Acute miliary TB single specified site |
| A19.1 | Acute miliary TB multiple sites |
| A19.2 | Acute miliary tuberculosis unspecified |
| A19.8 | Other miliary tuberculosis |
| A19.9 | Miliary tuberculosis unspecified |
| A20.0 | Bubonic plague |
| A20.1 | Cellulocutaneous plague |
| A20.2 | Pneumonic plague |
| A20.3 | Plague meningitis |
| A20.7 | Sepsis due to plague |
| A20.8 | Other forms of plague |
| A20.9 | Plague unspecified |
| A21.0 | Ulceroglandular tularaemia |
| A21.1 | Oculoglandular tularaemia |
| A21.2 | Pulmonary tularaemia |
| A21.3 | Gastrointestinal tularaemia |
| A21.7 | Sepsis due to tularaemia |
| A21.8 | Other forms of tularaemia |
| A21.9 | Tularaemia unspecified |
| A22.0 | Cutaneous anthrax |
| A22.1 | Pulmonary anthrax |
| A22.2 | Gastrointestinal anthrax |
| A22.7 | Sepsis due to anthrax |
| A22.8 | Other forms of anthrax |
| A22.9 | Anthrax unspecified |
| A23.0 | Brucellosis due to Brucella melitensis |
| A23.1 | Brucellosis due to Brucella abortus |
| A23.2 | Brucellosis due to Brucella suis |
| A23.3 | Brucellosis due to Brucella canis |
| A23.7 | Sepsis due to Brucella |
| A23.8 | Other brucellosis |
| A23.9 | Brucellosis unspecified |
| A24.0 | Glanders |
| A24.1 | Acute and fulminating melioidosis |
| A24.2 | Subacute and chronic melioidosis |
| A24.3 | Other melioidosis |
| A24.4 | Melioidosis unspecified |
| A24.7 | Sepsis due to glanders and melioidosis |
| A25.0 | Spirillosis |
| A25.1 | Streptobacillosis |
| A25.9 | Rat-bite fever unspecified |
| A26.0 | Cutaneous erysipeloid |
| A26.7 | Sepsis due to Erysipelothrix |
| A26.8 | Other forms of erysipeloid |
| A26.9 | Erysipeloid unspecified |
| A27.0 | Leptospirosis icterohaemorrhagica |
| A27.8 | Other forms of leptospirosis |
| A27.9 | Leptospirosis unspecified |
| A28.00 | Pasteurellosis not elsewhere classified |
| A28.01 | Sepsis due to Pasteurella NEC |
| A28.1 | Cat-scratch disease |
| A28.20 | Extraintestinal yersiniosis NEC |
| A28.21 | Sepsis dt extraintestinal yersiniosis |
| A28.8 | Other spec zoonotic bacterial dis NEC |
| A28.9 | Zoonotic bacterial disease unspecified |
| A31.0 | Pulmonary mycobacterial infection |
| A31.1 | Cutaneous mycobacterial infection |
| A31.8 | Other mycobacterial infections |
| A31.9 | Mycobacterial infection unspecified |
| A32.0 | Cutaneous listeriosis |
| A32.1 | Listerial meningitis meningoencephalitis |
| A32.7 | Sepsis due to Listeria monocytogenes |
| A32.8 | Other forms of listeriosis |
| A32.9 | Listeriosis unspecified |
| A35 | Other tetanus |
| A36.0 | Pharyngeal diphtheria |
| A36.1 | Nasopharyngeal diphtheria |
| A36.2 | Laryngeal diphtheria |
| A36.3 | Cutaneous diphtheria |
| A36.8 | Other diphtheria |
| A36.9 | Diphtheria unspecified |
| A37.0 | Whooping cough dt Bordetella pertussis |
| A37.1 | Whooping cough dt Bordetella parapertuss |
| A37.8 | Whooping cough dt oth Bordetella species |
| A37.9 | Whooping cough unspecified |
| A38 | Scarlet fever |
| A39.0 | Meningococcal meningitis |
| A39.1 | Waterhouse-Friderichsen syndrome |
| A39.2 | Acute meningococcaemia |
| A39.3 | Chronic meningococcaemia |
| A39.4 | Meningococcaemia unspecified |
| A39.5 | Meningococcal heart disease |
| A39.7 | Sepsis due to Meningococcus |
| A39.8 | Other meningococcal infections |
| A39.9 | Meningococcal infection unspecified |
| A40.0 | Sepsis dt Streptococcus group A |
| A40.1 | Sepsis dt Streptococcus group B |
| A40.21 | Sepsis due to Streptococcus group D |
| A40.22 | Sepsis due to Enterococcus |
| A40.3 | Sepsis dt Streptococcus pneumoniae |
| A40.8 | Other streptococcal sepsis |
| A40.9 | Streptococcal sepsis unspecified |
| A41.0 | Sepsis due to Staphylococcus aureus |
| A41.1 | Sepsis dt other spec Staphylococcus |
| A41.2 | Sepsis due to unsp Staphylococcus |
| A41.3 | Sepsis dt Haemophilus influenzae |
| A41.4 | Sepsis due to anaerobes |
| A41.50 | Sepsis dt unsp Gram neg organisms |
| A41.51 | Sepsis dt Escherichia coli [E coli] |
| A41.52 | Sepsis due to Pseudomonas |
| A41.58 | Sepsis dt other gram neg organisms |
| A41.8 | Sepsis due to other specified organism |
| A41.9 | Sepsis, unspecified |
| A42.0 | Pulmonary actinomycosis |
| A42.1 | Abdominal actinomycosis |
| A42.2 | Cervicofacial actinomycosis |
| A42.7 | Sepsis due to actinomycosis |
| A42.8 | Other forms of actinomycosis |
| A42.9 | Actinomycosis unspecified |
| A43.0 | Pulmonary nocardiosis |
| A43.1 | Cutaneous nocardiosis |
| A43.8 | Other forms of nocardiosis |
| A43.9 | Nocardiosis unspecified |
| A44.0 | Systemic bartonellosis |
| A44.1 | Cutaneous & mucocutaneous bartonellosis |
| A44.8 | Other forms of bartonellosis |
| A44.9 | Bartonellosis unspecified |
| A46 | Erysipelas |
| A48.0 | Gas gangrene |
| A48.1 | Legionnaires' disease |
| A48.2 | Nonpneumonic Legionnaires' disease |
| A48.3 | Toxic shock syndrome |
| A48.4 | Brazilian purpuric fever |
| A48.8 | Other specified bacterial diseases |
| A49.0 | Staphylococcal infection unspecified |
| A49.00 | Staphylococcal infection unspecified |
| A49.01 | Staphylococcus aureus infection unsp |
| A49.11 | Streptococcal infection unspecified site |
| A49.12 | Enterococcal infection unspecified site |
| A49.2 | Haemophilus influenzae infectn unsp site |
| A49.3 | Mycoplasma infection unsp site |
| A49.81 | Bacteroides fragilis infectn unsp site |
| A49.82 | Burkholderia infectn NEC unsp site |
| A49.83 | Campylobacter infectn unspecified site |
| A49.84 | Escherichia coli infection unsp site |
| A49.85 | K.pneumoniae infection unspecified site |
| A49.86 | Proteus Morganella & Providencia ? site |
| A49.87 | Pseudomonas infection unspecified site |
| A49.89 | Other bacterial infectn unspecified site |
| A49.9 | Bacterial infection unspecified |
| A51.0 | Primary genital syphilis |
| A51.1 | Primary anal syphilis |
| A51.2 | Primary syphilis of other sites |
| A51.3 | Sec syphilis of skin & mucous membranes |
| A51.4 | Other secondary syphilis |
| A51.5 | Early syphilis latent |
| A51.9 | Early syphilis unspecified |
| A52.0 | Cardiovascular syphilis |
| A52.1 | Symptomatic neurosyphilis |
| A52.2 | Asymptomatic neurosyphilis |
| A52.3 | Neurosyphilis unspecified |
| A52.7 | Other symptomatic late syphilis |
| A52.8 | Late syphilis latent |
| A52.9 | Late syphilis unspecified |
| A53.0 | Latent syphilis unsp as early or late |
| A53.9 | Syphilis unspecified |
| A54.0 | Low GU gon infectn wo abs acc periur gld |
| A54.1 | Low GU gon infectn w abs acc periur gld |
| A54.2 | Gon pelviperitonitis & oth gon GU infect |
| A54.3 | Gonococcal infection of eye |
| A54.4 | Gonococcal infectn musculoskeletal sys |
| A54.5 | Gonococcal pharyngitis |
| A54.6 | Gonococcal infection of anus and rectum |
| A54.7 | Sepsis due to Gonococcus |
| A54.8 | Other gonococcal infections |
| A54.9 | Gonococcal infection unspecified |
| A55 | Chlamydial lymphogranuloma (venereum) |
| A56.0 | Chlamydial infection lower GU tract |
| A56.1 | Chlamydial infectn pelviperit oth GU org |
| A56.2 | Chlamydial infectn GU tract unspecified |
| A56.3 | Chlamydial infection of anus and rectum |
| A56.4 | Chlamydial infection of pharynx |
| A56.8 | Sex trans chlamydial infectn other sites |
| A57 | Chancroid |
| A58 | Granuloma inguinale |
| A59.0 | Urogenital trichomoniasis |
| A59.8 | Trichomoniasis of other sites |
| A59.9 | Trichomoniasis unspecified |
| A60.0 | Herpesviral infectn genitalia & GU tract |
| A60.1 | Herpesviral infectn perianal skin rectum |
| A60.9 | Anogenital herpesviral infection unsp |
| A63.00 | Anogen (venereal) warts unspecified site |
| A63.01 | Perianal (venereal) warts |
| A63.02 | Cervical (venereal) warts |
| A63.03 | Urethral (venereal) warts |
| A63.04 | Vaginal (venereal) warts |
| A63.05 | Vulval (venereal) warts |
| A63.06 | Penile (venereal) warts |
| A63.07 | Scrotal (venereal) warts |
| A63.09 | Anogen (venereal) warts other site |
| A63.8 | Other spec predom sexually trans disease |
| A64 | Unspecified sexually transmitted disease |
| A65 | Nonvenereal syphilis |
| A66.0 | Initial lesions of yaws |
| A66.1 | Multiple papillomata and wet crab yaws |
| A66.2 | Other early skin lesions of yaws |
| A66.3 | Hyperkeratosis of yaws |
| A66.4 | Gummata and ulcers of yaws |
| A66.5 | Gangosa |
| A66.6 | Bone and joint lesions of yaws |
| A66.7 | Other manifestations of yaws |
| A66.8 | Latent yaws |
| A66.9 | Yaws unspecified |
| A67.0 | Primary lesions of pinta |
| A67.1 | Intermediate lesions of pinta |
| A67.2 | Late lesions of pinta |
| A67.3 | Mixed lesions of pinta |
| A67.9 | Pinta unspecified |
| A68.0 | Louse-borne relapsing fever |
| A68.1 | Tick-borne relapsing fever |
| A68.9 | Relapsing fever unspecified |
| A69.0 | Necrotising ulcerative stomatitis |
| A69.1 | Other Vincent's infections |
| A69.2 | Lyme disease |
| A69.8 | Other specified spirochaetal infections |
| A69.9 | Spirochaetal infection unspecified |
| A70 | Chlamydia psittaci infection |
| A71.0 | Initial stage of trachoma |
| A71.1 | Active stage of trachoma |
| A71.9 | Trachoma unspecified |
| A74.0 | Chlamydial conjunctivitis |
| A74.8 | Other chlamydial diseases |
| A74.9 | Chlamydial infection unspecified |
| A75.0 | Epi louse typhus dt Rickettsia prowazeki |
| A75.1 | Recrudescent typhus [Brill's disease] |
| A75.2 | Typhus fever due to Rickettsia typhi |
| A75.3 | Typhus fever dt Rickettsia tsutsugamushi |
| A75.9 | Typhus fever unspecified |
| A77.0 | Spotted fever dt Rickettsia rickettsii |
| A77.1 | Spotted fever due to Rickettsia conorii |
| A77.2 | Spotted fever due to Rickettsia siberica |
| A77.3 | Spotted fever dt Rickettsia australis |
| A77.8 | Other spotted fevers |
| A77.9 | Spotted fever unspecified |
| A78 | Q fever |
| A79.0 | Trench fever |
| A79.1 | Rickettsialpox due to Rickettsia akari |
| A79.8 | Other specified rickettsioses |
| A79.9 | Rickettsiosis unspecified |
| A83.0 | Creutzfeldt-Jakob disease |
| A83.1 | Subacute sclerosing panencephalitis |
| A83.2 | Progress multifocal leukoencephalopathy |
| A83.3 | Other atypical viral infections of CNS |
| A83.4 | Atypical virus infection of CNS unsp |
| A83.9 | Japanese encephalitis |
| A84.0 | Western equine encephalitis |
| A84.1 | Eastern equine encephalitis |
| A84.8 | St Louis encephalitis |
| A84.9 | Australian encephalitis |
| A85.0 | California encephalitis |
| A85.1 | Rocio virus disease |
| A85.2 | Other mosquito-borne viral encephalitis |
| A85.8 | Mosquito borne encephalitis unsp |
| A86 | Far Eastern tick-borne encephalitis |
| A87.0 | Central European tick-borne encephalitis |
| A87.1 | Other tick-borne viral encephalitis |
| A87.2 | Tick-borne encephalitis unsp |
| A87.8 | Enteroviral encephalitis |
| A87.9 | Adenoviral encephalitis |
| A88.0 | Arthropod-borne viral encephalitis unsp |
| A88.1 | Other specified viral encephalitis |
| A88.8 | Unspecified viral encephalitis |
| A89 | Enteroviral meningitis |
| A92.0 | Adenoviral meningitis |
| A92.1 | Lymphocytic choriomeningitis |
| A92.2 | Other viral meningitis |
| A92.3 | Viral meningitis unspecified |
| A92.4 | Enteroviral exanthematous fever |
| A92.5 | Epidemic vertigo |
| A92.8 | Other specified viral infections of CNS |
| A92.9 | Unspecified viral infection of CNS |
| B00.0 | Eczema herpeticum |
| B00.1 | Herpesviral vesicular dermatitis |
| B00.2 | Herpes gingivostomatis pharyngotonsillit |
| B00.3 | Herpesviral meningitis |
| B00.4 | Herpesviral encephalitis |
| B00.5 | Herpesviral ocular disease |
| B00.70 | Disseminated herpesviral disease NEC |
| B00.71 | Sepsis due to herpesviral infection |
| B00.8 | Other forms of herpesviral infection |
| B00.9 | Herpesviral infection unspecified |
| B01.0 | Varicella meningitis |
| B01.1 | Varicella encephalitis |
| B01.2 | Varicella pneumonia |
| B01.8 | Varicella with other complications |
| B01.9 | Varicella without complication |
| B02.0 | Zoster encephalitis |
| B02.1 | Zoster meningitis |
| B02.2 | Zoster w oth nervous system involvement |
| B02.3 | Zoster ocular disease |
| B02.7 | Disseminated zoster |
| B02.8 | Zoster with other complications |
| B02.9 | Zoster without complication |
| B03 | Smallpox |
| B04 | Monkeypox |
| B05.0 | Measles complicated by encephalitis |
| B05.1 | Measles complicated by meningitis |
| B05.2 | Measles complicated by pneumonia |
| B05.3 | Measles complicated by otitis media |
| B05.4 | Measles with intestinal complications |
| B05.8 | Measles with other complications |
| B05.9 | Measles without complication |
| B06.0 | Rubella with neurological complications |
| B06.8 | Rubella with other complications |
| B06.9 | Rubella without complication |
| B07 | Viral warts |
| B08.0 | Other orthopoxvirus infections |
| B08.1 | Molluscum contagiosum |
| B08.2 | Exanthema subitum [sixth disease] |
| B08.3 | Erythema infectiosum [fifth disease] |
| B08.4 | Entervir vesicular stomatitis w exanthem |
| B08.5 | Enteroviral vesicular pharyngitis |
| B08.8 | Oth viral infections w skin & mucous lsn |
| B09 | Unsp viral infectn w skin & mucous lsn |
| B15.0 | Hepatitis A with hepatic coma |
| B15.9 | Hepatitis A without hepatic coma |
| B16.0 | Ac hep B w delta-agent w hepatic coma |
| B16.1 | Ac hep B w delta-agent wo hepatic coma |
| B16.2 | Ac hep B wo delta-agent w hepatic coma |
| B16.9 | Ac hep B wo delta-agent wo hepatic coma |
| B17.0 | Ac delta (super) infectn chr hep B |
| B17.1 | Acute hepatitis C |
| B17.2 | Acute hepatitis E |
| B17.8 | Other specified acute viral hepatitis |
| B17.9 | Acute viral hepatitis unspecified |
| B18.0 | Chronic viral hep B w delta agent |
| B18.1 | Chronic viral hep B wo delta agent |
| B18.2 | Chronic viral hepatitis C |
| B18.8 | Other chronic viral hepatitis |
| B18.9 | Chronic viral hepatitis unspecified |
| B19.0 | Unsp viral hepatitis with hepatic coma |
| B19.9 | Unsp viral hepatitis wo hepatic coma |
| B20 | HIV resulting in infect & parasitic dis |
| B21 | HIV resulting in malignant neoplasms |
| B22 | HIV resulting in other spec diseases |
| B23.0 | Acute HIV infection syndrome |
| B23.8 | HIV resulting in other spec conditions |
| B24 | Unspecified HIV disease |
| B25.0 | Cytomegaloviral pneumonitis |
| B25.1 | Cytomegaloviral hepatitis |
| B25.2 | Cytomegaloviral pancreatitis |
| B25.8 | Other cytomegaloviral diseases |
| B25.9 | Cytomegaloviral disease unspecified |
| B26.0 | Mumps orchitis |
| B26.1 | Mumps meningitis |
| B26.2 | Mumps encephalitis |
| B26.3 | Mumps pancreatitis |
| B26.8 | Mumps with other complications |
| B26.9 | Mumps without complication |
| B27.0 | Gammaherpesviral mononucleosis |
| B27.1 | Cytomegaloviral mononucleosis |
| B27.8 | Other infectious mononucleosis |
| B27.9 | Infectious mononucleosis unspecified |
| B30.0 | Keratoconjunctivitis dt adenovirus |
| B30.1 | Conjunctivitis dt adenovirus |
| B30.2 | Viral pharyngoconjunctivitis |
| B30.3 | Acute epidemic haem conjunctivitis |
| B30.8 | Other viral conjunctivitis |
| B30.9 | Viral conjunctivitis unspecified |
| B33.0 | Epidemic myalgia |
| B33.1 | Ross River disease |
| B33.2 | Viral carditis |
| B33.3 | Retrovirus infections NEC |
| B33.4 | Hantavirus (cardio-) pulmonary syndrome |
| B33.8 | Other specified viral diseases |
| B34.0 | Adenovirus infection unspecified site |
| B34.1 | Enterovirus infection unspecified site |
| B34.2 | Coronavirus infection unspecified site |
| B34.3 | Parvovirus infection unspecified site |
| B34.4 | Papovavirus infection unspecified site |
| B34.8 | Other viral infections of unsp site |
| B34.9 | Viral infection unspecified |
| B35.0 | Tinea barbae and tinea capitis |
| B35.1 | Tinea unguium |
| B35.2 | Tinea manuum |
| B35.3 | Tinea pedis |
| B35.4 | Tinea corporis |
| B35.5 | Tinea imbricata |
| B35.6 | Tinea inguinalis [tinea cruris] |
| B35.8 | Other dermatophytoses |
| B35.9 | Dermatophytosis unspecified |
| B36.0 | Pityriasis versicolor |
| B36.1 | Tinea nigra |
| B36.2 | White piedra |
| B36.3 | Black piedra |
| B36.8 | Other specified superficial mycoses |
| B36.9 | Superficial mycosis unspecified |
| B37.0 | Candidal stomatitis |
| B37.1 | Pulmonary candidiasis |
| B37.2 | Candidiasis of skin and nail |
| B37.3 | Candidiasis of vulva and vagina |
| B37.4 | Candidiasis of other urogenital sites |
| B37.5 | Candidal meningitis |
| B37.6 | Candidal endocarditis |
| B37.7 | Sepsis due to Candida |
| B37.81 | Candidal oesophagitis |
| B37.82 | Candida albicans |
| B37.83 | Candida auris |
| B37.89 | Candidiasis of other sites |
| B37.9 | Candidiasis unspecified site |
| B38.0 | Acute pulmonary coccidioidomycosis |
| B38.1 | Chronic pulmonary coccidioidomycosis |
| B38.2 | Pulmonary coccidioidomycosis unsp |
| B38.3 | Cutaneous coccidioidomycosis |
| B38.4 | Coccidioidomycosis meningitis |
| B38.7 | Disseminated coccidioidomycosis |
| B38.8 | Other forms of coccidioidomycosis |
| B38.9 | Coccidioidomycosis unspecified |
| B39.0 | Acute pulmonary histoplasmosis capsulati |
| B39.1 | Chr pulmonary histoplasmosis capsulati |
| B39.2 | Pulmonary histoplasmosis capsulati unsp |
| B39.3 | Disseminated histoplasmosis capsulati |
| B39.4 | Histoplasmosis capsulati unspecified |
| B39.5 | Histoplasmosis duboisii |
| B39.9 | Histoplasmosis unspecified |
| B40.0 | Acute pulmonary blastomycosis |
| B40.1 | Chronic pulmonary blastomycosis |
| B40.2 | Pulmonary blastomycosis unspecified |
| B40.3 | Cutaneous blastomycosis |
| B40.7 | Disseminated blastomycosis |
| B40.8 | Other forms of blastomycosis |
| B40.9 | Blastomycosis unspecified |
| B41.0 | Pulmonary paracoccidioidomycosis |
| B41.7 | Disseminated paracoccidioidomycosis |
| B41.8 | Other forms of paracoccidioidomycosis |
| B41.9 | Paracoccidioidomycosis unspecified |
| B42.0 | Pulmonary sporotrichosis |
| B42.1 | Lymphocutaneous sporotrichosis |
| B42.7 | Disseminated sporotrichosis |
| B42.8 | Other forms of sporotrichosis |
| B42.9 | Sporotrichosis unspecified |
| B43.0 | Cutaneous chromomycosis |
| B43.1 | Phaeomycotic brain abscess |
| B43.2 | Subcutaneous phaeomycotic abscess & cyst |
| B43.8 | Other forms of chromomycosis |
| B43.9 | Chromomycosis unspecified |
| B44.0 | Invasive pulmonary aspergillosis |
| B44.1 | Other pulmonary aspergillosis |
| B44.2 | Tonsillar aspergillosis |
| B44.7 | Disseminated aspergillosis |
| B44.8 | Other forms of aspergillosis |
| B44.9 | Aspergillosis unspecified |
| B45.0 | Pulmonary cryptococcosis |
| B45.1 | Cerebral cryptococcosis |
| B45.2 | Cutaneous cryptococcosis |
| B45.3 | Osseous cryptococcosis |
| B45.7 | Disseminated cryptococcosis |
| B45.8 | Other forms of cryptococcosis |
| B45.9 | Cryptococcosis unspecified |
| B46.0 | Pulmonary mucormycosis |
| B46.1 | Rhinocerebral mucormycosis |
| B46.2 | Gastrointestinal mucormycosis |
| B46.3 | Cutaneous mucormycosis |
| B46.4 | Disseminated mucormycosis |
| B46.5 | Mucormycosis unspecified |
| B46.8 | Other zygomycoses |
| B46.9 | Zygomycosis unspecified |
| B47.0 | Eumycetoma |
| B47.1 | Actinomycetoma |
| B47.9 | Mycetoma unspecified |
| B48.0 | Lobomycosis |
| B48.1 | Rhinosporidiosis |
| B48.2 | Allescheriasis |
| B48.3 | Geotrichosis |
| B48.4 | Penicilliosis |
| B48.5 | Pneumocystosis |
| B48.7 | Opportunistic mycoses |
| B48.8 | Other specified mycoses |
| B49 | Unspecified mycosis |
| B90.0 | Sequelae of CNS tuberculosis |
| B90.1 | Sequelae of genitourinary tuberculosis |
| B90.2 | Sequelae of tuberculosis bones & joints |
| B90.8 | Sequelae of tuberculosis of other organs |
| B90.9 | Sequelae of respiratory & unspecified TB |
| B94.0 | Sequelae of trachoma |
| B94.1 | Sequelae of viral encephalitis |
| B94.2 | Sequelae of viral hepatitis |
| B94.8 | Seq of other spec infect & parasitic dis |
| B94.9 | Seq of unsp infect & parasitic dis |
| B95.0 | Grp A strep cause dis class to oth chptr |
| B95.1 | Grp B strep cause dis class to oth chptr |
| B95.21 | Strep grp D cause dis class oth chptr |
| B95.22 | Enterococcus cause dis class oth chptr |
| B95.3 | Strep pneum caus dis class oth chptr |
| B95.41 | Grp C strep cause dis class to oth chptr |
| B95.42 | Grp G strep cause dis class to oth chptr |
| B95.48 | Strep spec grp cause dis class oth chptr |
| B95.5 | Unsp strep cause dis class to oth chptr |
| B95.6 | Staph aureus cause dis class oth chptr |
| B95.71 | Staph argenteus cause dis oth chptr |
| B95.79 | Other staph cause dis other chapter |
| B95.8 | Unsp staph cause dis class to oth chptr |
| B96.0 | M. pneumoniae cause dis class oth chptr |
| B96.1 | K. pneumoniae cause dis class oth chptr |
| B96.2 | E coli cause dis class to oth chptr |
| B96.31 | H. influenzae type B cause dis oth chptr |
| B96.38 | H. influenzae spec causing dis oth chptr |
| B96.39 | H. influenzae unsp cause dis oth chptr |
| B96.41 | Proteus cause dis class to oth chptr |
| B96.42 | Morganella cause dis class to oth chptr |
| B96.43 | Providencia cause dis class to oth chptr |
| B96.5 | Pseudomonas cause dis class to oth chptr |
| B96.6 | Bacteroides cause dis class to oth chptr |
| B96.7 | C. perfringens cause dis class oth chptr |
| B96.81 | H. pylori causing dis oth chptr |
| B96.82 | Vibrio vulnificus cause dis oth chptr |
| B96.83 | A. baumannii cause dis class oth chptr |
| B96.84 | Burkholderia cause dis class oth chptr |
| B96.85 | Campylobacter cause dis class oth chptr |
| B96.86 | C.difficile cause dis class oth chptr |
| B96.87 | Oth enterobacterales cause dis oth chptr |
| B96.89 | Oth spec bact agt cause dis oth chptr |
| B97.0 | Adenovirus cause dis class to oth chptr |
| B97.1 | Enterovirus cause dis class to oth chptr |
| B97.2 | Coronavirus cause dis class to oth chptr |
| B97.3 | Retrovirus cause dis class to oth chptr |
| B97.4 | Resp syncytial virus cause dis oth chptr |
| B97.5 | Reovirus cause dis class to oth chptr |
| B97.6 | Parvovirus cause dis class to oth chptr |
| B97.7 | Papillomavirus cause dis class oth chptr |
| B97.81 | Rhinovirus cause dis class oth chptr |
| B97.82 | Astrovirus cause dis class oth chptr |
| B97.83 | Parainfluenza cause dis class oth chptr |
| B97.89 | Viral agt cause dis class oth chptr NEC |
| B99 | Other & unspecified infectious diseases |
| ENDOCRINE, NUTRITIONAL AND METABOLIC DISEASES | |
| E32.1 | Abscess of thymus |
| DISEASES OF THE NERVOUS SYSTEM | |
| G00.0 | Haemophilus meningitis |
| G00.1 | Pneumococcal meningitis |
| G00.2 | Streptococcal meningitis NEC |
| G00.3 | Staphylococcal meningitis |
| G00.8 | Other bacterial meningitis |
| G00.9 | Bacterial meningitis unspecified |
| G01 | Meningitis in bact dis class elsewhere |
| G02.0 | Meningitis in viral dis class elsewhere |
| G02.1 | Meningitis in mycoses |
| G02.8 | Mengits oth spec infect parasit dis cl/e |
| G03.0 | Nonpyogenic meningitis |
| G03.1 | Chronic meningitis |
| G03.2 | Benign recurrent meningitis [Mollaret] |
| G03.8 | Meningitis due to other specified causes |
| G03.9 | Meningitis unspecified |
| G04.0 | Acute disseminated encephalitis |
| G04.1 | HTLV associated myelopathy |
| G04.2 | Bact meningoencephalit meningomyelit NEC |
| G04.8 | Oth encephalit myelitis encephalomyelit |
| G04.9 | Encephalit myelitis encephalomyelit unsp |
| G05.0 | Encephalit &/or myelitis bact dis cl/e |
| G05.1 | Encephalit &/or myelitis viral dis cl/e |
| G05.2 | Encephalit &or myelit infect parasit dis |
| G05.8 | Encephalit &/or myelitis oth dis cl/e |
| G06.0 | Intracranial abscess and granuloma |
| G06.1 | Intraspinal abscess and granuloma |
| G06.2 | Extradural & subdural abscess unsp |
| G07 | Intrcran intraspinal abs granuloma cl/e |
| G08 | Intrcran intraspin phleb & thrombophleb |
| DISEASES OF THE EYE AND ADNEXA | |
| H05.0 | Acute inflammation of orbit |
| H44.0 | Purulent endophthalmitis |
| H44.1 | Other endophthalmitis |
| H45.1 | Endophthalmitis in diseases cl/e |
| H60.0 | Abscess of external ear |
| H62.0 | Otitis externa in bacterial disease cl/e |
| H62.1 | Otitis externa in viral disease cl/e |
| H62.2 | Otitis externa in mycoses |
| H62.3 | Otitis ext oth infect parasit dis cl/e |
| H62.4 | Otitis externa in other diseases cl/e |
| H66.0 | Acute suppurative otitis media |
| H67.0 | Otitis media in bacterial diseases cl/e |
| H67.1 | Otitis media in viral diseases cl/e |
| H67.8 | Otitis media in other diseases cl/e |
| H70.0 | Acute mastoiditis |
| H75.0 | Mastoiditis in infect & parasit dis cl/e |
| DISEASES OF THE CIRCULATORY SYSTEM | |
| I30.1 | Infective pericarditis |
| I32.0 | Pericarditis in bact dis class elsewhere |
| I32.1 | Pericarditis oth infect parasit dis cl/e |
| I33.0 | Acute & subacute infective endocarditis |
| I33.9 | Acute endocarditis unspecified |
| I40.0 | Infective myocarditis |
| I41.0 | Myocarditis in bacterial diseases cl/e |
| I41.1 | Myocarditis in viral diseases cl/e |
| I41.2 | Myocarditis in infect & parasit dis cl/e |
| I43.0 | Cardiomyopathy infect parasit dis cl/e |
| I68.1 | Cereb arteritis infect parasit dis cl/e |
| I80.0 | Phleb & thrombophleb spfl vesl legs |
| I80.1 | Phlebitis & thrombophleb femoral vein |
| I80.20 | Phleb & thrombophleb deep low extrem NEC |
| I80.21 | Phleb & thrombophleb iliac vein |
| I80.22 | Phleb & thrombophleb popliteal vein |
| I80.23 | Phleb & thrombophleb tibial vein |
| I80.3 | Phlebitis & thrombophlebitis legs unsp |
| I80.40 | Phleb & thrombophleb upp extrem NEC |
| I80.41 | Phleb & thrombophleb spfl upp extrem |
| I80.42 | Phleb & thrombophleb deep upp extrem |
| I80.8 | Phlebitis & thrombophlebitis other sites |
| I80.9 | Phlebitis & thrombophlebitis unsp site |
| I98.0 | Cardiovascular syphilis |
| I98.1 | C-V disrd in oth infect parasit dis cl/e |
| DISEASES OF THE RESPIRATORY SYSTEM | |
| J00 | Acute nasopharyngitis |
| J01.0 | Acute maxillary sinusitis |
| J01.1 | Acute frontal sinusitis |
| J01.2 | Acute ethmoidal sinusitis |
| J01.3 | Acute sphenoidal sinusitis |
| J01.4 | Acute pansinusitis |
| J01.8 | Other acute sinusitis |
| J01.9 | Acute sinusitis unspecified |
| J02.0 | Streptococcal pharyngitis |
| J02.8 | Acute pharyngitis dt oth spec organisms |
| J02.9 | Acute pharyngitis unspecified |
| J03.0 | Streptococcal tonsillitis |
| J03.8 | Acute tonsillitis dt oth spec organisms |
| J03.9 | Acute tonsillitis unspecified |
| J04.0 | Acute laryngitis |
| J04.1 | Acute tracheitis |
| J04.2 | Acute laryngotracheitis |
| J05.0 | Acute obstructive laryngitis [croup] |
| J05.1 | Acute epiglottitis |
| J06.0 | Acute laryngopharyngitis |
| J06.8 | Other acute URTI of multiple sites |
| J06.9 | Acute URTI unspecified |
| J09 | Influenza dt id zoo pand influenza virus |
| J10.0 | Influenza w pneumonia other virus id |
| J10.1 | Influenza w oth resp manif oth virus id |
| J10.8 | Influenza w oth manif other virus id |
| J11.0 | Influenza w pneum virus not identified |
| J11.1 | Influenza w oth resp manif virus not id |
| J11.8 | Influenza w oth manif virus not id |
| J12.0 | Adenoviral pneumonia |
| J12.1 | Respiratory syncytial virus pneumonia |
| J12.2 | Parainfluenza virus pneumonia |
| J12.3 | Human metapneumovirus pneumonia |
| J12.8 | Other viral pneumonia |
| J12.9 | Viral pneumonia unspecified |
| J13 | Pneumonia dt Streptococcus pneumoniae |
| J14 | Pneumonia due to Haemophilus influenzae |
| J15.0 | Pneumonia due to Klebsiella pneumoniae |
| J15.1 | Pneumonia due to Pseudomonas |
| J15.2 | Pneumonia due to Staphylococcus |
| J15.3 | Pneumonia due to Streptococcus group B |
| J15.4 | Pneumonia due to other streptococci |
| J15.5 | Pneumonia due to Escherichia coli |
| J15.6 | Pneumonia dt oth gram neg bact |
| J15.7 | Pneumonia dt Mycoplasma pneumoniae |
| J15.8 | Other bacterial pneumonia |
| J15.9 | Bacterial pneumonia unspecified |
| J16.0 | Chlamydial pneumonia |
| J16.1 | Fungal pneumonia NEC |
| J16.8 | Pneumonia dt oth spec infect organisms |
| J17.0 | Pneumonia in bact dis class elsewhere |
| J17.1 | Pneumonia in viral dis class elsewhere |
| J17.2 | Pneumonia in mycoses |
| J17.3 | Pneumonia in parasitic diseases |
| J17.8 | Pneumonia in other dis class elsewhere |
| J18.0 | Bronchopneumonia unspecified |
| J18.1 | Lobar pneumonia unspecified |
| J18.2 | Hypostatic pneumonia unspecified |
| J18.8 | Other pneumonia organism unspecified |
| J18.9 | Pneumonia unspecified |
| J20.0 | Ac bronchitis dt Mycoplasma pneumoniae |
| J20.1 | Ac bronchitis dt Haemophilus influenzae |
| J20.2 | Acute bronchitis due to Streptococcus |
| J20.3 | Acute bronchitis due to coxsackievirus |
| J20.4 | Acute bronchitis dt parainfluenza virus |
| J20.5 | Ac bronchitis dt resp syncytial virus |
| J20.6 | Acute bronchitis due to rhinovirus |
| J20.7 | Acute bronchitis due to echovirus |
| J20.8 | Acute bronchitis dt other spec organisms |
| J20.9 | Acute bronchitis unspecified |
| J21.0 | Ac bronchiolitis dt resp syncytial virus |
| J21.1 | Ac brchlts dt human metapneumovirus |
| J21.8 | Ac bronchiolitis dt other spec organisms |
| J21.9 | Acute bronchiolitis unspecified |
| J22 | Unsp acute lower respiratory infection |
| J32.0 | Chronic maxillary sinusitis |
| J34.0 | Abscess furuncle and carbuncle of nose |
| J35.0 | Chronic tonsillitis |
| J36 | Peritonsillar abscess |
| J37.0 | Chronic laryngitis |
| J39.0 | Retropharyngeal & parapharyngeal abscess |
| J39.1 | Other abscess of pharynx |
| J41.0 | Simple chronic bronchitis |
| J41.1 | Mucopurulent chronic bronchitis |
| J41.2 | Protracted bacterial bronchitis |
| J41.8 | Mx simple & mucopurulent chr bronchitis |
| J42 | Unspecified chronic bronchitis |
| J44.0 | COPD with acute lower resp infection |
| J44.1 | COPD with acute exacerbation unspecified |
| J85.0 | Gangrene and necrosis of lung |
| J85.1 | Abscess of lung with pneumonia |
| J85.2 | Abscess of lung without pneumonia |
| J85.3 | Abscess of mediastinum |
| J86.0 | Pyothorax with fistula |
| J86.9 | Pyothorax without fistula |
| J99.8 | Respiratory disrd in other diseases cl/e |
| DISEASES OF THE DIGESTIVE SYSTEM | |
| K04.6 | Periapical abscess with sinus |
| K04.7 | Periapical abscess without sinus |
| K05.2 | Acute periodontitis |
| K05.3 | Chronic periodontitis |
| K11.3 | Abscess of salivary gland |
| K12.2 | Cellulitis and abscess of mouth |
| K35.0 | Ac appendicitis w genl peritonitis |
| K35.1 | Ac appendicitis w peritoneal abcess |
| K35.2 | Ac appendicitis w genl peritonitis |
| K35.3 | Ac appendicitis w localised peritonitis |
| K35.8 | Ac appendicitis oth & unspecified |
| K35.9 | Acute appendicitis, unspecified |
| K36 | Other appendicitis |
| K37 | Unspecified appendicitis |
| K57.02 | Diverlitis sm intest w perf abs wo haem |
| K57.03 | Diverlitis sm intest w haem perf & abs |
| K57.20 | Divertic lrg intest w perf & abs wo haem |
| K57.21 | Divertic large intest w haem perf & abs |
| K57.22 | Diverlitis lrg intest w perf abs wo haem |
| K57.23 | Diverlitis lrg intest w haem perf & abs |
| K57.40 | Divertic both intest w perf abs wo haem |
| K57.41 | Divertic both intest w haem perf & abs |
| K57.42 | Diverlitis bth intest w perf abs wo haem |
| K57.43 | Diverlitis both intest w haem perf & abs |
| K57.82 | Diverlitis intest ? w perf abs wo haem |
| K57.83 | Diverlitis intest unsp w haem perf & abs |
| K61.0 | Anal abscess |
| K61.1 | Rectal abscess |
| K61.2 | Anorectal abscess |
| K61.3 | Ischiorectal abscess |
| K61.4 | Intrasphincteric abscess |
| K63.0 | Abscess of intestine |
| K63.1 | Perforation of intestine (nontraumatic) |
| K65.11 | Spontaneous bacterial peritonitis |
| K65.19 | Primary peritonitis NEC |
| K65.21 | Eosinophilic peritonitis |
| K65.22 | Mesenteric peritonitis |
| K65.23 | Chronic proliferative peritonitis |
| K65.24 | Chemical peritonitis |
| K65.29 | Secondary peritonitis NEC |
| K65.3 | Peritoneal abscess |
| K65.8 | Other peritonitis |
| K65.9 | Peritonitis unspecified |
| K67.0 | Chlamydial peritonitis (A74.8+) |
| K67.1 | Gonococcal peritonitis (A54.8+) |
| K67.2 | Syphilitic peritonitis (A52.7+) |
| K67.3 | Tuberculous peritonitis (A18.3+) |
| K75.0 | Abscess of liver |
| K75.1 | Phlebitis of portal vein |
| K77.0 | Liver disrd in infect & parasit dis cl/e |
| K80.00 | Calculus gallb w ac cholecystitis wo obs |
| K81.0 | Acute cholecystitis |
| K83.0 | Cholangitis |
| K87.1 | Disrd pancreas in dis class elsewhere |
| K93.0 | Tuberculous disorders of intestines |
| DISEASES OF THE SKIN AND SUBCUTANEOUS TISSUE | |
| L03.01 | Cellulitis of finger |
| L03.02 | Cellulitis of toe |
| L03.12 | Cellulitis of upper limb |
| L03.13 | Cellulitis of lower limb |
| L03.14 | Cellulitis of foot |
| L03.19 | Cellulitis of limb NEC |
| L03.2 | Cellulitis of face |
| L03.3 | Cellulitis of trunk |
| L03.8 | Cellulitis of other sites |
| L03.9 | Cellulitis unspecified |
| L04.0 | Acute lymphadenitis face head & neck |
| L04.1 | Acute lymphadenitis of trunk |
| L04.2 | Acute lymphadenitis of upper limb |
| L04.3 | Acute lymphadenitis of lower limb |
| L04.8 | Acute lymphadenitis of other sites |
| L04.9 | Acute lymphadenitis unspecified |
| L08.0 | Pyoderma |
| L08.1 | Erythrasma |
| L08.8 | Oth spec local infectn skin sbc tissue |
| DISEASES OF THE MUSCULOSKELETAL SYSTEM AND CONNECTIVE TISSUE | |
| M00.00 | Staph arthritis polyarthritis mult sites |
| M00.01 | Staph arthritis & polyarthritis shoulder |
| M00.02 | Staph arthritis polyarthritis upper arm |
| M00.03 | Staph arthritis & polyarthritis forearm |
| M00.04 | Staph arthritis & polyarthritis hand |
| M00.05 | Staph arthr polyarthr pelv rgn & thgh |
| M00.06 | Staph arthritis polyarthritis lower leg |
| M00.07 | Staph arthritis polyarthritis ankle foot |
| M00.08 | Staph arthritis polyarthritis other site |
| M00.09 | Staph arthritis polyarthritis site unsp |
| M00.10 | Pneumcoc arthritis polyarthr mult sites |
| M00.11 | Pneumcoc arthritis polyarthr shoulder |
| M00.12 | Pneumcoc arthritis polyarthr upper arm |
| M00.13 | Pneumcoc arthritis polyarthritis forearm |
| M00.14 | Pneumcoc arthritis polyarthritis hand |
| M00.15 | Pneumcoc arthr polyarthr pelv rgn & thgh |
| M00.16 | Pneumcoc arthritis polyarthr lower leg |
| M00.17 | Pneumcoc arthritis polyarthr ankle foot |
| M00.18 | Pneumcoc arthritis polyarthritis other |
| M00.19 | Pneumcoc arthritis polyarthr site unsp |
| M00.20 | Oth strep arthritis polyarthr mult sites |
| M00.21 | Oth strep arthritis polyarthr shoulder |
| M00.22 | Oth strep arthritis polyarthr upper arm |
| M00.23 | Oth strep arthritis polyarthr forearm |
| M00.24 | Oth strep arthritis polyarthritis hand |
| M00.25 | Oth strep arthritis polyarthr pelv thigh |
| M00.26 | Oth strep arthritis polyarthr lower leg |
| M00.27 | Oth strep arthritis polyarthr ankle foot |
| M00.28 | Oth strep arthritis polyarthritis other |
| M00.29 | Oth strep arthritis polyarthr site unsp |
| M00.80 | Arthritis polyarthr dt oth bact mult sit |
| M00.81 | Arthritis polyarthr dt oth bact shoulder |
| M00.82 | Arthritis polyarthr dt oth bact upp arm |
| M00.83 | Arthritis polyarthr dt oth bact forearm |
| M00.84 | Arthritis polyarthritis dt oth bact hand |
| M00.85 | Arthr polyarthr dt oth bact pelv thigh |
| M00.86 | Arthritis polyarthr dt oth bact low leg |
| M00.87 | Arthr polyarthr dt oth bact ankle foot |
| M00.88 | Arthritis polyarthr dt oth spec bact oth |
| M00.89 | Arthr polyarthr dt oth spec bact site ? |
| M00.90 | Pyogenic arthritis unsp mult sites |
| M00.91 | Pyogenic arthritis unsp shoulder |
| M00.92 | Pyogenic arthritis unsp upper arm |
| M00.93 | Pyogenic arthritis unspecified forearm |
| M00.94 | Pyogenic arthritis unspecified hand |
| M00.95 | Pyogenic arthritis unsp pelv rgn & thgh |
| M00.96 | Pyogenic arthritis unsp lower leg |
| M00.97 | Pyogenic arthritis unsp ankle & foot |
| M00.98 | Pyogenic arthritis unspecified other |
| M00.99 | Pyogenic arthritis unspecified site unsp |
| M49.30 | Spondlpth oth infect dis mult site spin |
| M60.00 | Infective myositis multiple sites |
| M63.00 | Myositis in bact dis cl/e mult sites |
| M63.01 | Myositis in bact dis cl/e shoulder rgn |
| M63.02 | Myositis in bact dis cl/e upper arm |
| M63.03 | Myositis in bacterial dis cl/e forearm |
| M63.04 | Myositis in bacterial dis cl/e hand |
| M63.05 | Myosit in bact dis cl/e pelv rgn & thgh |
| M63.06 | Myositis in bact dis cl/e lower leg |
| M63.07 | Myositis in bact dis cl/e ankle & foot |
| M63.08 | Myositis in bacterial dis cl/e other |
| M63.09 | Myositis in bacterial dis cl/e site unsp |
| M63.10 | Myosit protzl parasit infectn cl/e mult |
| M63.11 | Myosit protzl parasit infectn cl/e shold |
| M63.12 | Myosit protzl parasit infectn upp arm |
| M63.13 | Myosit protzl parasit infectn forearm |
| M63.14 | Myosit protzl parasit infectn cl/e hand |
| M63.15 | Myosit protzl parasit infectn pelv thigh |
| M63.16 | Myosit protzl parasit infectn low leg |
| M63.17 | Myosit protzl parasit infectn ankle ft |
| M63.18 | Myosit protzl parasit infectn cl/e other |
| M63.19 | Myosit protzl parasit infectn site unsp |
| M63.20 | Myositis in oth infect dis cl/e mult sit |
| M63.21 | Myositis in oth infect dis cl/e shoulder |
| M63.22 | Myositis in oth infect dis cl/e upp arm |
| M63.23 | Myositis in oth infect dis cl/e forearm |
| M63.24 | Myositis in oth infect dis cl/e hand |
| M63.25 | Myositis oth infect dis cl/e pelv thgh |
| M63.26 | Myositis in oth infect dis cl/e low leg |
| M63.27 | Myositis in oth infect dis cl/e ankle ft |
| M63.28 | Myositis in other infect dis cl/e other |
| M63.29 | Myositis in oth infect dis cl/e sit unsp |
| M65.00 | Abscess of tendon sheath multiple sites |
| M65.10 | Oth infective (teno)synovitis mult sites |
| M71.00 | Abscess of bursa multiple sites |
| M71.10 | Other infective bursitis multiple sites |
| M72.60 | Necrotising fasciitis mult sites |
| M73.00 | Gonococcal bursitis mult sites (A54.4+) |
| M73.10 | Syphilitic bursitis mult sites (A52.7+) |
| M73.80 | Oth soft tis disrd in dis cl/e mult site |
| M86.0 | Acute haematogenous osteomyelitis |
| M86.00 | Ac haematogenous osteomyelitis mult site |
| M86.01 | Ac haematogenous osteomyelitis shoulder |
| M86.02 | Ac haematogenous osteomyelitis upp arm |
| M86.03 | Ac haematogenous osteomyelitis forearm |
| M86.04 | Acute haematogenous osteomyelitis hand |
| M86.05 | Ac haematogenous osteomyelitis pelv thgh |
| M86.06 | Ac haematogenous osteomyelitis low leg |
| M86.07 | Ac haematogenous osteomyelitis ankle ft |
| M86.08 | Acute haematogenous osteomyelitis other |
| M86.09 | Ac haematogenous osteomyelitis site unsp |
| M86.1 | Other acute osteomyelitis |
| M86.10 | Other acute osteomyelitis mult sites |
| M86.11 | Other acute osteomyelitis shoulder |
| M86.12 | Other acute osteomyelitis upper arm |
| M86.13 | Other acute osteomyelitis forearm |
| M86.14 | Other acute osteomyelitis hand |
| M86.15 | Oth acute osteomyelitis pelv rgn & thgh |
| M86.16 | Other acute osteomyelitis lower leg |
| M86.17 | Other acute osteomyelitis ankle & foot |
| M86.18 | Other acute osteomyelitis other |
| M86.19 | Other acute osteomyelitis site unsp |
| M66.2 | Subacute osteomyelitis |
| M86.20 | Subacute osteomyelitis multiple sites |
| M86.21 | Subacute osteomyelitis shoulder region |
| M86.22 | Subacute osteomyelitis upper arm |
| M86.23 | Subacute osteomyelitis forearm |
| M86.24 | Subacute osteomyelitis hand |
| M86.25 | Subacute osteomyelitis pelv rgn & thgh |
| M86.26 | Subacute osteomyelitis lower leg |
| M86.27 | Subacute osteomyelitis ankle and foot |
| M86.28 | Subacute osteomyelitis other |
| M86.29 | Subacute osteomyelitis site unspecified |
| M86.3 | Chronic multifocal osteomyelitis |
| M86.30 | Chr multifocal osteomyelitis mult sites |
| M86.31 | Chr multifocal osteomyelitis shoulder |
| M86.32 | Chr multifocal osteomyelitis upper arm |
| M86.33 | Chr multifocal osteomyelitis forearm |
| M86.34 | Chronic multifocal osteomyelitis hand |
| M86.35 | Chr multifocal osteomyelitis pelv thgh |
| M86.36 | Chr multifocal osteomyelitis lower leg |
| M86.37 | Chr multifocal osteomyelitis ankle foot |
| M86.38 | Chronic multifocal osteomyelitis other |
| M86.39 | Chr multifocal osteomyelitis site unsp |
| M86.40 | Chr osteomyelitis w drain sinus mult sit |
| M86.41 | Chr osteomyelitis w drain sinus shoulder |
| M86.42 | Chr osteomyelitis w drain sinus upp arm |
| M86.43 | Chr osteomyelitis w drain sinus forearm |
| M86.44 | Chr osteomyelitis w draining sinus hand |
| M86.45 | Chr osteomyelitis drain sinus pelv thgh |
| M86.46 | Chr osteomyelitis w drain sinus low leg |
| M86.47 | Chr osteomyelitis w drain sinus ankle ft |
| M86.48 | Chr osteomyelitis w drain sinus other |
| M86.49 | Chr osteomyelts w drain sinus site unsp |
| M65.5 | Other chronic haematogenous osteomyelitis |
| M86.50 | Oth chr haemtgs osteomyelitis mult site |
| M86.51 | Oth chr haemtgs osteomyelitis shoulder |
| M86.52 | Oth chr haemtgs osteomyelitis upper arm |
| M86.53 | Oth chr haemtgs osteomyelitis forearm |
| M86.54 | Oth chr haematogenous osteomyelitis hand |
| M86.55 | Oth chr haemtgs osteomyelitis pelv thgh |
| M86.56 | Oth chr haemtgs osteomyelitis low leg |
| M86.57 | Oth chr haemtgs osteomyelitis ankle ft |
| M86.58 | Oth chr haemtgs osteomyelitis other |
| M86.59 | Oth chr haemtgs osteomyelitis site unsp |
| M86.60 | Other chronic osteomyelitis mult sites |
| M86.61 | Other chronic osteomyelitis shoulder |
| M86.62 | Other chronic osteomyelitis upper arm |
| M86.63 | Other chronic osteomyelitis forearm |
| M86.64 | Other chronic osteomyelitis hand |
| M86.65 | Oth chr osteomyelitis pelv rgn & thgh |
| M86.66 | Other chronic osteomyelitis lower leg |
| M86.67 | Other chronic osteomyelitis ankle foot |
| M86.68 | Other chronic osteomyelitis other |
| M86.69 | Other chronic osteomyelitis site unsp |
| M68.8 | Other osteomyelitis |
| M86.80 | Other osteomyelitis multiple sites |
| M86.81 | Other osteomyelitis shoulder region |
| M86.82 | Other osteomyelitis upper arm |
| M86.83 | Other osteomyelitis forearm |
| M86.84 | Other osteomyelitis hand |
| M86.85 | Other osteomyelitis pelv rgn & thgh |
| M86.86 | Other osteomyelitis lower leg |
| M86.87 | Other osteomyelitis ankle and foot |
| M86.88 | Other osteomyelitis other |
| M86.89 | Other osteomyelitis site unspecified |
| M869 | Osteomyelitis, unspecified |
| M86.90 | Osteomyelitis unspecified mult sites |
| M86.91 | Osteomyelitis unspecified shoulder |
| M86.92 | Osteomyelitis unspecified upper arm |
| M86.93 | Osteomyelitis unspecified forearm |
| M86.94 | Osteomyelitis unspecified hand |
| M86.95 | Osteomyelitis unsp pelv rgn & thgh |
| M86.96 | Osteomyelitis unspecified lower leg |
| M86.97 | Osteomyelitis unspecified ankle & foot |
| M86.98 | Osteomyelitis unspecified other site |
| M86.99 | Osteomyelitis unspecified site unsp |
| M90.00 | Tuberculosis of bone (A18.0+) mult sites |
| M90.10 | Periostitis oth infect dis cl/e mult sit |
| M90.20 | Osteopathy oth infect dis cl/e mult site |
| DISEASES OF THE GENITOURINARY SYSTEM | |
| N10 | Acute tubulo-interstitial nephritis |
| N11.0 | Nonobstr reflux-ass chronic pyeloneph |
| N11.1 | Chronic obstructive pyelonephritis |
| N11.8 | Oth chr tubulo-interstitial nephritis |
| N11.9 | Chr tubulo-interstitial nephritis unsp |
| N12 | Tubulo-interstitial nephritis NOS |
| N15.1 | Renal and perinephric abscess |
| N16.0 | Ren tubulointerst disrd infect dis cl/e |
| N30.0 | Acute cystitis |
| N33.0 | Tuberculous cystitis (A18.1+) |
| N34.0 | Urethral abscess |
| N34.1 | Nonspecific urethritis |
| N34.2 | Other urethritis |
| N41.0 | Acute prostatitis |
| N41.1 | Chronic prostatitis |
| N41.2 | Abscess of prostate |
| N41.3 | Prostatocystitis |
| N41.8 | Other inflammatory diseases of prostate |
| N41.9 | Inflammatory disease of prostate unsp |
| N45.0 | Orchitis epididymitis w abscess |
| N48.1 | Balanoposthitis |
| N70.0 | Acute salpingitis and oophoritis |
| N70.1 | Chronic salpingitis and oophoritis |
| N70.9 | Salpingitis and oophoritis unspecified |
| N71.0 | Acute inflammatory disease of uterus |
| N71.1 | Chronic inflammatory disease of uterus |
| N71.9 | Inflammatory disease of uterus unsp |
| N72 | Inflammatory disease of cervix uteri |
| N73.0 | Acute parametritis and pelvic cellulitis |
| N73.1 | Chronic parametritis & pelvic cellulitis |
| N73.2 | Unsp parametritis and pelvic cellulitis |
| N73.3 | Female acute pelvic peritonitis |
| N73.4 | Female chronic pelvic peritonitis |
| N73.5 | Female pelvic peritonitis unspecified |
| N73.6 | Female pelvic peritoneal adhesions |
| N73.8 | Oth spec female pelvic inflammatory dis |
| N73.9 | Female pelvic inflammatory disease unsp |
| N74.0 | Tuberculous infection cervix uteri |
| N74.1 | Female tuberculous pelvic inf dis |
| N74.2 | Female syphilitic pelvic inf dis |
| N74.3 | Female gonococcal pelvic inf dis |
| N74.4 | Female chlamydial pelvic inf dis |
| N74.8 | Female pelvic inf disrd in oth dis cl/e |
| N75.0 | Cyst of Bartholin's gland |
| N75.1 | Abscess of Bartholin's gland |
| N75.8 | Other diseases of Bartholin's gland |
| N75.9 | Disease of Bartholin's gland unsp |
| N76.0 | Acute vaginitis |
| N76.1 | Subacute and chronic vaginitis |
| N76.2 | Acute vulvitis |
| N76.3 | Subacute and chronic vulvitis |
| N76.4 | Abscess of vulva |
| N76.5 | Ulceration of vagina |
| N76.6 | Ulceration of vulva |
| N76.8 | Oth spec inflammation of vagina & vulva |
| N77.0 | Ulceration vulva infect parasit dis cl/e |
| N77.1 | Vaginits vulvits infect parasit dis cl/e |
| SYMPTOMS, SIGNS AND ABNORMAL CLINICAL AND LABORATORY FINDINGS, NOT ELSEWHERE CLASSIFIED | |
| R50.8 | Other specified fever |
| R50.9 | Fever unspecified |
| R57.2 | Septic shock |
| INJURY, POISONING AND CERTAIN OTHER CONSEQUENCES OF EXTERNAL CAUSES | |
| T80.2 | Infection foll infus transfn thrpc injct |
| T81.4 | Wound infection following a proc NEC |
| T81.41 | Wound infection following a procedure |
| T81.42 | Sepsis following a procedure |
| T82.6 | Infectn inf react dt card valve prosth |
| T82.7 | Infection and inflammatory reaction due to other cardiac and vascular prosthetic devices, implants and grafts |
| T82.71 | Infectn inf dt electronic card dev |
| T82.72 | Infectn inf dt coron art byps & grafts |
| T82.73 | Infectn inf dt oth vasc grafts |

Source: ICD-10-AM Chronicle – First Edition to Eleventh Edition
